# Sparse and Simple Structure Estimation via Prenet Penalization

**DOI:** 10.1007/s11336-022-09868-4

**Published:** 2022-05-23

**Authors:** Kei Hirose, Yoshikazu Terada

**Affiliations:** 1 Institute of Mathematics for Industry, Kyushu University, Fukuoka, Japan; 2 Graduate School of Engineering Science, Osaka University, Osaka, Japan; 3 RIKEN Center for Advanced Intelligence Project, Wakō, Japan

**Keywords:** multivariate analysis, quartimin rotation, penalized maximum likelihood estimation, perfect simple structure, sparse estimation

## Abstract

We propose a *prenet* (*pr*oduct-based *e*lastic *net*), a novel penalization method for factor analysis models. The penalty is based on the product of a pair of elements in each row of the loading matrix. The prenet not only shrinks some of the factor loadings toward exactly zero but also enhances the simplicity of the loading matrix, which plays an important role in the interpretation of the common factors. In particular, with a large amount of prenet penalization, the estimated loading matrix possesses a perfect simple structure, which is known as a desirable structure in terms of the simplicity of the loading matrix. Furthermore, the perfect simple structure estimation via the proposed penalization turns out to be a generalization of the *k*-means clustering of variables. On the other hand, a mild amount of the penalization approximates a loading matrix estimated by the quartimin rotation, one of the most commonly used oblique rotation techniques. Simulation studies compare the performance of our proposed penalization with that of existing methods under a variety of settings. The usefulness of the perfect simple structure estimation via our proposed procedure is presented through various real data applications.

**Supplementary Information:**

The online version contains supplementary material available at 10.1007/s11336-022-09868-4.

Factor analysis investigates the correlation structure of high-dimensional observed variables by the construction of a small number of latent variables called common factors. Factor analysis can be considered as a soft clustering of variables, in which each factor corresponds to a cluster and observed variables are categorized into overlapping clusters. For interpretation purposes, it is desirable for the observed variables to be well-clustered (Yamamoto and Jennrich, 2013) or the loading matrix to be simple (Thurstone, 1947). In particular, the perfect simple structure (e.g., Bernaards and Jennrich, 2003; Jennrich, 2004), wherein each row of the loading matrix has at most one nonzero element, provides a non-overlapping clustering of variables in the sense that variables that correspond to nonzero elements of the *j*th column of the loading matrix belong to the *j*th cluster.

Conventionally, the well-clustered or simple structure of the loading matrix is found by rotation techniques. A number of rotation techniques have been proposed in the literature; for example, quartimin rotation (Carroll, 1953), varimax rotation (Kaiser, 1958), promax rotation (Hendrickson and White, 1964), simplimax rotation (Kiers, 1994), geomin rotation (Yates, 1987), and component loss criterion (Jennrich, 2004, 2006). The literature review of the rotation techniques is described in Browne 2001. The main purpose of the factor rotation is to get a good solution that is as simple as possible. See, e.g., Thurstone (1935), Carroll (1953), Neuhaus and Wrigley (1954), Kaiser (1974), Bernaards and Jennrich (2003).

The problem with the rotation technique is that it cannot produce a sufficiently sparse solution in some cases (Hirose and Yamamoto, 2015), because the loading matrix must be found among a set of unpenalized maximum likelihood estimates. To obtain sparser solutions than the factor rotation, we employ a penalization method. It is shown that the penalization is a generalization of the rotation techniques and can produce sparser solutions than the rotation methods (Hirose and Yamamoto, 2015). Typically, many researchers use the $L_1$-type penalization, such as the lasso (Tibshirani, 1996), the adaptive lasso (Zou, 2006), and the minimax concave penalty (MCP) (Zhang, 2010); for example, Choi et al. (2011), Ning and Georgiou (2011), Srivastava et al. (2017), Hirose and Yamamoto (2015), Trendafilov et al. (2017), Hui et al. (2018). The $L_1$ penalization shrinks some of the parameters toward exactly zero; in other words, parameters that need not to be modeled are automatically disregarded. Furthermore, the degrees of sparsity are freely adjusted by changing the value of a regularization parameter. The $L_1$ penalization provides a good estimation accuracy, such as $L_1$ consistency and model selection consistency in high dimension (e.g., Zhao and Yu 2007; Wainwright, 2009; Bhlmann and van de Geer, 2011).

As described above, it is important to obtain a “good” loading matrix in the sense of simplicity and thus interpretability in the exploratory factor analysis. Although the $L_1$ penalization achieves the sparse estimation, the estimated loading matrix is not guaranteed to possess an interpretable structure. For example, a great amount of penalization leads to a zero matrix, which does not make sense from an interpretation viewpoint. Thus, the $L_1$ penalization cannot produce an interpretable loading matrix with a sufficient large regularization parameter. Even if a small value of regularization parameter is selected, the $L_1$ penalization cannot often approximate a true loading matrix when it is not sufficiently sparse; with the lasso, some of the factor loadings whose true values are close —but not very close—to zero are estimated as zero values, and this misspecification can often cause a significant negative effect on the estimation of other factor loadings (Hirose and Yamamoto, 2014). Therefore, it is important to estimate a loading matrix that is not only sparse but also interpretable. To achieve this, we need a different type of penalty.

In this study, we propose a *prenet* (*pr*oduct-based *e*lastic *net*) penalty, which is based on the product of a pair of parameters in each row of the loading matrix. A remarkable feature of the prenet is that a large amount of penalization leads to the perfect simple structure. The existing $L_1$-type penalization methods do not have that significant property. Furthermore, the perfect simple structure estimation via the prenet penalty is shown to be a generalization of the *k*-means clustering of variables. On the other hand, with a mild amount of prenet penalization, the estimated loading matrix is approximated by that obtained using the quartimin rotation, a widely used oblique rotation method. The quartimin criterion can often estimate a non-sparse loading matrix appropriately, so that the problem of the lasso-type penalization mentioned above is addressed. We employ the generalized expectation and maximization (EM) algorithm and the coordinate descent algorithm (e.g., Friedman et al., 2010) to obtain the estimator. The proposed algorithm monotonically decreases the objective function at each iteration.

In our proposed procedure, the regularization parameter controls the degrees of simplicity; the larger the regularization parameter is, the simpler the loading matrix is. The advantage of our proposed procedure is that we can change the degrees of simplicity according to the purpose of the analysis. This study focus on two different purposes of the analysis: (i) to find a loading matrix that fits the data and also is simple as much as possible and (ii) to conduct cluster analysis by estimating a perfect simple structure. The regularization parameter selection procedure differs depending on these two purposes. To achieve the purpose (i), we select the regularization parameter by the Akaike information criterion (AIC; Akaike, 1973; Zou et al., 2007) or the Bayesian information criterion (BIC; Schwarz, 1978; Zou et al., 2007). The purpose (ii) is attained by setting the regularization parameter to be infinity.

We conduct the Monte Carlo simulations to compare the performance of our proposed method with that of $L_1$-type penalization and conventional rotation techniques. The Monte Carlo simulations investigate the performance in terms of both (i) and (ii); investigations of (i) and (ii) are detailed in Sects. 5.2 and 5.3, respectively. Our proposed method is applied to data from big five personality traits to study the performance for various sample sizes and impact of the regularization parameter on the accuracy. The analysis of big five personality traits aims at purpose (i). We also present the analyses of fMRI and electricity demand data, intended to purpose both (i) and (ii), in Section S2 of the supplemental material.

The rest of this article is organized as follows. Section 1 describes the estimation of the factor analysis model via penalization. In Sect. 2, we introduce the prenet penalty. Section 3 describes several properties of the prenet penalty, including its relationship with the quartimin criterion. Section 4 presents an estimation algorithm to obtain the prenet solutions. In Sect. 5, we conduct a Monte Carlo simulation to investigate the performance of the prenet penalization. Section 6 illustrates the usefulness of our proposed procedure through real data analysis. Extension and future works are discussed in Sect. 7. Some technical proofs and detail of our algorithm are shown in “Appendix.” Supplemental materials include further numerical and theoretical investigation, including numerical analyses of resting-state fMRI and electricity demand data.

## Estimation of Factor Analysis Model via Penalization

Let $\varvec{X} = (X_1,\dots ,X_p)^T$ be a *p*-dimensional observed random vector with mean vector $\varvec{0}$ and variance–covariance matrix $\varvec{\Sigma }$. The factor analysis model is

1 $$\begin{aligned} \varvec{X} = \varvec{\Lambda } \varvec{F}+\varvec{\varepsilon }, \end{aligned}$$

where $\varvec{\Lambda } = (\lambda _{ij})$ is a $p \times m$ loading matrix, $\varvec{F} = (F_1,\ldots ,F_m)^T$ is a random vector of common factors, and $\varvec{\varepsilon } = (\varepsilon _1,\ldots , \varepsilon _p)^T$ is a random vector of unique factors. It is assumed that ${E}(\varvec{F} ) = \varvec{0}$, ${E}(\varvec{\varepsilon } ) = \varvec{0}$, ${E}(\varvec{F}\varvec{F}^T) = \varvec{\Phi }$, ${E}(\varvec{\varepsilon } \varvec{\varepsilon } ^T) = \varvec{\Psi }$, and ${E}(\varvec{F} \varvec{\varepsilon } ^T) = \varvec{O}$, where $\varvec{\Phi }$ is an $m \times m$ factor correlation matrix, and $\varvec{\Psi }$ is a $p \times p$ diagonal matrix (i.e., strict factor model). The diagonal elements of $\varvec{\Psi }$ are referred to as unique variances. Under these assumptions, the variance–covariance matrix of observed random vector $\varvec{X}$ is $\varvec{\Sigma } = \varvec{\Lambda } \varvec{\Phi }\varvec{\Lambda }^T+\varvec{\Psi }$.

In many cases, the orthogonal factor model (i.e., $\varvec{\Phi } = \varvec{I}_m$) is used but is often oversimplified. Here, $\varvec{I}_m$ is an identity matrix of order *m*. This paper covers the oblique factor model, which allows a more realistic estimation of latent factors than the orthogonal factor model in many cases (e.g., Fabrigar et al., 1999; Sass and Schmitt, 2010; Schmitt and Sass, 2011).

Let $\varvec{x}_1,\ldots ,\varvec{x}_n$ be *n* observations and $\varvec{S} = (s_{ij})$ be the corresponding sample covariance matrix. Let $\varvec{\theta } = (\mathrm{vec}(\varvec{\Lambda })^T,\mathrm{diag}(\varvec{\Psi })^T,\mathrm{vech}(\varvec{\Phi })^T)^T$ be a parameter vector, where vech$(\cdot )$ is a vector that consists of a lower triangular matrix without diagonal elements. We estimate the model parameter by minimizing the penalized loss function $\ell _{\rho }(\varvec{\theta })$ expressed as

2 $$\begin{aligned} \ell _{\rho }(\varvec{\theta }) = \ell (\varvec{\theta }) + \rho P(\varvec{\Lambda }), \end{aligned}$$

where $\ell (\varvec{\theta })$ is a loss function, $P(\varvec{\Lambda })$ is a penalty function, and $\rho > 0$ is a regularization parameter. As a loss function, we adopt the maximum likelihood discrepancy function

3 $$\begin{aligned} \ell _{\text {DF}}(\varvec{\theta }) = \frac{1}{2} \left\{ \mathrm {tr}(\varvec{\Sigma }^{-1} \varvec{S}) - \log |\varvec{\Sigma }^{-1}\varvec{S}| - p \right\} , \end{aligned}$$

where DF is an abbreviation for discrepancy function. Assume that the observations $\varvec{x}_1,\ldots ,\varvec{x}_n$ are drawn from the *p*-dimensional normal population $N_p(\varvec{0},\varvec{\Sigma })$ with $\varvec{\Sigma } = \varvec{\Lambda }\varvec{\Phi } \varvec{\Lambda }^T+\varvec{\Psi }$. The minimizer of $\ell _{\text {DF}}(\varvec{\theta })$ is the maximum likelihood estimate. It is shown that $\ell _{\text {DF}}(\varvec{\theta })\ge 0$ for any $\varvec{\theta }$, and $\ell _{\text {DF}}(\varvec{\theta }) = 0$ if and only if $\varvec{\Lambda }\varvec{\Phi }\varvec{\Lambda }^T + \varvec{\Psi } = \varvec{S}$ when $\varvec{\Sigma }$ and $\varvec{S}$ are positive definite matrices. It is worth noting that our proposed penalty, described in Sect. 2, can be directly applied to many other loss functions, including a quadratic loss used for generalized least squares (Jöreskog and Goldberger, 1971).

When $\varvec{\Phi }=\varvec{I}_m$, the model has a rotational indeterminacy; both $\varvec{\Lambda }$ and $\varvec{\Lambda } \varvec{T}$ generate the same covariance matrix $\varvec{\Sigma }$, where $\varvec{T}$ is an arbitrary orthogonal matrix. Thus, when $\rho = 0$, the solution that minimizes (2) is not uniquely determined. However, when $\rho > 0$, the solution may be uniquely determined except for the sign and permutation of columns of the loading matrix when an appropriate penalty $P(\varvec{\Lambda })$ is chosen.

## Prenet Penalty

### Definition

We propose a prenet penalty

4 $$\begin{aligned} P(\varvec{\Lambda }) = \sum _{i = 1}^p \sum _{j = 1}^{m-1} \sum _{k > j} \left\{ \gamma |\lambda _{ij}\lambda _{ik}| + \frac{1}{2} (1-\gamma ) (\lambda _{ij}\lambda _{ik})^2 \right\} , \end{aligned}$$

where $\gamma \in (0,1]$ is a tuning parameter. The most significant feature of the prenet penalty is that it is based on the product of a pair of parameters.

When $\gamma \rightarrow 0$, the prenet penalty is equivalent to the quartimin criterion (Carroll, 1953), a widely used oblique rotation criterion in factor rotation. As is the case with the quartimin rotation, the prenet penalty in (4) eliminates the rotational indeterminacy except for the sign and permutation of columns of the loading matrix and contributes significantly to the estimation of the simplicity of the loading matrix. The prenet penalty includes products of absolute values of factor loadings, producing factor loadings that are exactly zero.

### Comparison with the Elastic Net Penalty

The prenet penalty is similar to the elastic net penalty (Zou and Hastie, 2005)

5 $$\begin{aligned} P(\varvec{\Lambda }) = \sum _{i = 1}^p \sum _{j = 1}^{m} \left\{ \gamma |\lambda _{ij}| + \frac{1}{2} (1-\gamma ) \lambda _{ij}^2 \right\} , \end{aligned}$$

which is a hybrid of the lasso and the ridge penalties. Although the elastic net penalty is similar to the prenet penalty, there is a fundamental difference between these two penalties; the elastic net is constructed by the sum of the elements of parameter vector, whereas the prenet is based on the product of a pair of parameters.

**Fig. 1:**
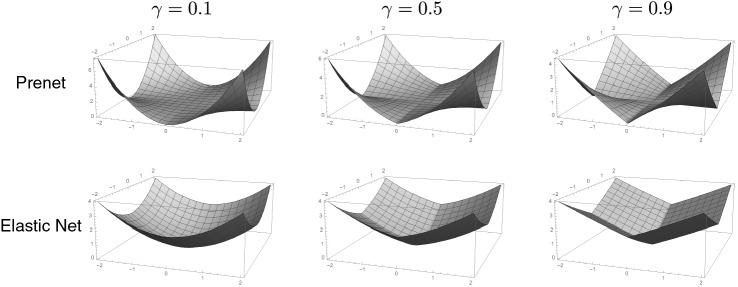
Penalty functions of the prenet and the elastic net with various $\gamma$.

Figure 1 shows the penalty functions of the prenet ($P(x,y) = \gamma |xy| + (1-\gamma )(xy)^2/2$) and the elastic net ($P(x,y) = \gamma (|x|+|y|) + (1-\gamma )(x^2+y^2)/2$) when $\gamma =0.1$, 0.5, 0.9. Clearly, the prenet penalty is a nonconvex function. A significant difference between the prenet and the elastic net is that although the prenet penalty becomes zero when either *x* or *y* attains zero, the elastic net penalty becomes zero only when both $x = 0$ and $y = 0$. Therefore, for a two-factor model, the estimate of either $\lambda _{i1}$ or $\lambda _{i2}$ can be zero with the prenet penalization, leading to a perfect simple structure. On the other hand, the elastic net tends to produce estimates in which both $|\lambda _{i1}|$ and $|\lambda _{i2}|$ are small.

The penalty functions in Fig. 1 also show that the prenet penalty becomes smooth as $\gamma$ decreases. Thus, the value of $\gamma$ controls the degrees of sparsity; the larger the value of $\gamma$, the sparser the estimate of the loading matrix. With an appropriate value of $\gamma$, the prenet penalty enhances both simplicity and sparsity of the loading matrix. Further investigation into the estimator against the value of $\gamma$ is presented in Sect. 5.

## Properties of the Prenet Penalty

### Perfect Simple Structure

The model (1) does not impose an orthogonal constraint on the loading matrix $\varvec{\Lambda }$. For unconstrained $\varvec{\Lambda }$, most existing penalties, such as the lasso, shrink all coefficients toward zero when the tuning parameter $\rho$ is sufficiently large; we usually obtain $\hat{\varvec{\Lambda }} = \varvec{O}$ when $\rho \rightarrow \infty$. However, the following proposition shows that the prenet penalty does not necessarily shrink all of the elements toward zero even when $\rho$ is sufficiently large.

#### Proposition 1

Assume that we use the prenet penalty with $\gamma \in (0,1]$. As $\rho \rightarrow \infty$, the estimated loading matrix possesses the perfect simple structure, that is, each row has at most one nonzero element.

#### Proof

As $\rho \rightarrow \infty$, $P(\hat{\varvec{\Lambda }})$ must satisfy $P(\hat{\varvec{\Lambda }}) \rightarrow 0$. Otherwise, the second term of (2) diverges. When $P(\hat{\varvec{\Lambda }}) = 0$, ${\hat{\lambda }}_{ij}{\hat{\lambda }}_{ik} = 0$ for any $j \ne k$. Therefore, the *i*th row of $\varvec{\Lambda }$ has at most one nonzero element. $\square$

The perfect simple structure is known as a desirable property in the literature in factor analysis because it is easy to interpret the estimated loading matrix (e.g., Bernaards and Jennrich, 2003). When $\rho$ is small, the estimated loading matrix can be away from the perfect simple structure but the goodness of fit to the model is improved.

#### Remark 1

For $\rho \rightarrow \infty$, the consistency of the loading matrix is shown when the true loading matrix possesses the perfect simple structure. For simplicity, we consider the orthogonal case. Assume that $\varvec{\Sigma } = \varvec{\Lambda }\varvec{\Lambda }^T + \varvec{\Psi }$, where the true $\varvec{\Lambda }$ possesses the perfect simple structure. As $n \rightarrow \infty$, the sample covariance matrix converges to the true covariance matrix almost surely; thus, the loss function (3) is minimized when $\varvec{\Sigma } = \hat{\varvec{\Lambda }}\hat{\varvec{\Lambda }}^T + \hat{\varvec{\Psi }}$. When $\rho \rightarrow \infty$, $\hat{\varvec{\Lambda }}$ must be the perfect simple structure. Therefore, we should consider the following problem:

$$\begin{aligned} \text{ Find } (\hat{\varvec{\Lambda }}, \hat{\varvec{\Psi }}) \text{ that } \text{ satisfies } \left\{ \begin{array}{l} \varvec{\Sigma } = \hat{\varvec{\Lambda }}\hat{\varvec{\Lambda }}^T + \hat{\varvec{\Psi }}, \\ \hat{\varvec{\Lambda }} \text{ is } \text{ perfect } \text{ simple } \text{ structure. } \end{array} \right. \end{aligned}$$

The solution to the above problem is $\hat{\varvec{\Lambda }} = \varvec{\Lambda }$ except for the sign and permutation of columns of the loading matrix if an identifiability condition for an orthogonal model (e.g., Theorem 5.1 in Anderson and Rubin, 1956) is satisfied.

### Relationship with *k*-Means Clustering of Variables

The perfect simple structure corresponds to variables clustering, that is, variables that correspond to nonzero elements of the *j*th column of the loading matrix belong to the *j*th cluster. In this subsection, we investigate the relationship between the prenet with $\rho \rightarrow \infty$ and the *k*-means clustering of variables, one of the most popular cluster analyses.

Let $\varvec{X}_0$ be an $n \times p$ data matrix. $\varvec{X}_0$ can be expressed as $\varvec{X}_0 = (\varvec{x}_1^*,\dots ,\varvec{x}_p^*)$, where $\varvec{x}_i^*$ is the *i*th column vector of $\varvec{X}_0$. We consider the problem of the variables clustering of $\varvec{x}_1^*,\dots ,\varvec{x}_p^*$ by the *k*-means. Let $C_j$ $(j = 1,\dots ,m)$ be a subset of indices of variables that belong to the *j*th cluster. The objective function of the *k*-means is

6 $$\begin{aligned} \sum _{j = 1}^m \sum _{i \in C_j}\Vert \varvec{x}_i^* - \varvec{\mu }_j\Vert ^2 = (n-1) \left( \sum _{i = 1}^ps_{ii} - \sum _{j=1}^m \frac{1}{p_j}\sum _{i \in C_j}\sum _{i' \in C_j}s_{ii'} \right) , \end{aligned}$$

where $p_j = \#\{C_j\}$, $\varvec{\mu }_j = \frac{1}{p_j}\sum _{i \in C_j} \varvec{x}_i^*$, and recall that $s_{ii'} = \varvec{x}_i^{*T}\varvec{x}_{i'}^*/(n-1)$. Let $\varvec{\Lambda } = (\lambda _{ij})$ be a $p \times m$ indicator variables matrix

7 $$\begin{aligned} \lambda _{ij} = \left\{ \begin{array}{ll} 1/\sqrt{p_j} &{} i \in C_j, \\ 0 &{} i \notin C_j. \end{array} \right. \end{aligned}$$

Using the fact that $\varvec{\Lambda }^T\varvec{\Lambda } = \varvec{I}_m$, the *k*-means clustering of variables using (6) is equivalent to (Ding et al., 2005)

8 $$\begin{aligned} \min _{\varvec{\Lambda }} \Vert \varvec{S} - \varvec{\Lambda }\varvec{\Lambda }^T\Vert _{F}^2, \ \text{ subject } \text{ to } (7), \end{aligned}$$

where $\Vert \cdot \Vert _F$ denotes the Frobenius norm. We consider slightly modifying the condition on $\varvec{\Lambda }$ in (7) to

9 $$\begin{aligned} \lambda _{ij} \lambda _{ik} = 0 \ (j \ne k) \text{ and } \varvec{\Lambda }^T\varvec{\Lambda } = \varvec{I}_m. \end{aligned}$$

The modified *k*-means problem is then given as

10 $$\begin{aligned} \min _{\varvec{\Lambda }} \Vert \varvec{S} - \varvec{\Lambda }\varvec{\Lambda }^T\Vert _{F}^2 \text{ subject } \text{ to } (9). \end{aligned}$$

The condition (9) is milder than (7); if $\varvec{\Lambda }$ satisfies (7), we obtain (9). The reverse does not always hold; with (9), the nonzero elements for each column do not have to be equal. Therefore, the modified *k*-means in (10) may capture a more complex structure than the original *k*-means.

#### Proposition 2

Assume that $\varvec{\Psi } = \alpha \varvec{I}_p$, $\varvec{\Phi }=\varvec{I}_m$, and $\alpha$ is given. Suppose that $\varvec{\Lambda }$ satisfies $\varvec{\Lambda }^T\varvec{\Lambda } = \varvec{I}_m$. The prenet solution with $\rho \rightarrow \infty$ is then obtained by (10).

#### Proof

The proof appears in Appendix A.1. $\square$

The proposition 2 shows that the prenet solution with $\rho \rightarrow \infty$ is a generalization of the problem (10). As mentioned above, the problem (10) is a generalization of the *k*-means problem in (8). Therefore, the perfect simple structure estimation via the prenet is a generalization of the *k*-means clustering of variables. We remark that the condition $\varvec{\Psi } = \alpha \varvec{I}_p$ in Proposition 2 implies the probabilistic principal component analysis (probabilistic PCA; Tipping and Bishop, 1999); the penalized probabilistic PCA via the prenet is also a generalization of the *k*-means clustering of variables.

### Relationship with Quartimin Rotation

As described in Sect. 2, the prenet penalty is a generalization of the quartimin criterion (Carroll, 1953); setting $\gamma \rightarrow 0$ to the prenet penalty in (4) leads to the quartimin criterion

$$\begin{aligned} P_{\mathrm{qmin}}(\varvec{\Lambda }) = \sum _{i = 1}^p \sum _{j = 1}^{m-1 } \sum _{k > j} (\lambda _{ij}\lambda _{ik})^2. \end{aligned}$$

The quartimin criterion is typically used in the factor rotation. The solution of quartimin rotation method, say $\hat{\varvec{\theta }}_q$, is obtained by two-step procedure. First, we calculate an unpenalized estimate, denoted by $\hat{\varvec{\theta }}$. The estimate $\hat{\varvec{\theta }}$, that satisfies $\displaystyle {\ell }(\hat{\varvec{\theta }})=\min _{\varvec{\theta }} {\ell }(\varvec{\theta })$, is not unique due to the rotational indeterminacy. The second step is the minimization of the quartimin criterion with a restricted parameter space $\{ \varvec{\theta }|\ell (\varvec{\theta }) = \min _{\varvec{\theta }}\ell (\varvec{\theta }) \}$. Hirose and Yamamoto (2015) showed that the solution of the quartimin rotation, $\hat{\varvec{\theta }}_q$, can be obtained by

11 $$\begin{aligned} \min _{\varvec{\theta }}P_{\mathrm{qmin}}(\varvec{\Lambda }), \text{ subject } \text{ to } \quad \ell (\varvec{\theta }) = \ell (\hat{\varvec{\theta }}) \end{aligned}$$

under the condition that the unpenalized estimate of loading matrix $\hat{\varvec{\Lambda } }$ is unique if the indeterminacy of the rotation in $\hat{\varvec{\Lambda } }$ is excluded. It is not easy to check this condition, but several necessary conditions of the identifiability for the orthogonal model are provided (e.g., Theorem 5.1 in Anderson and Rubin, 1956.)

Now, we show a basic asymptotic result of the prenet solution, from which we can see that the prenet solution is a generalization of the quartimin rotation. Let $(\Theta ,d)$ be a compact parameter space with distance *d* and $(\Omega ,{\mathcal {F}},{\mathbb {P}})$ be a probability space. Suppose that for any $(\mathrm{vec}(\varvec{\Lambda })^T,\mathrm{diag}(\varvec{\Psi })^T,\mathrm{vech}(\varvec{\Phi })^T)^T\in \Theta$ and any $\varvec{T}\in {\mathcal {O}}(m)$, we have $(\mathrm{vec}(\varvec{\Lambda }\varvec{T})^T,\mathrm{diag}(\varvec{\Psi })^T,\mathrm{vech}(\varvec{\Phi })^T)^T\in \Theta$, where ${\mathcal {O}}(m)$ is a set of $m\times m$ orthonormal matrices. Let $\varvec{X}_1,\dots ,\varvec{X}_n$ denote independent ${\mathbb {R}}^p$-valued random variables with the common population distribution ${\mathbb {P}}$. Now, it is required that we can rewrite the empirical loss function and the true loss function as $\ell (\varvec{\theta })=\sum _{i=1}^n q(\varvec{X}_i;\varvec{\theta })/n$ and $\ell _*(\varvec{\theta })=\int q(\varvec{x};\varvec{\theta })\,{\mathbb {P}}(d\varvec{x})$, respectively. Let $\hat{\varvec{\theta }}_\rho$ denote an arbitrary measurable prenet estimator which satisfies $\ell (\hat{\varvec{\theta }}_\rho ) +\rho P(\hat{\varvec{\Lambda }}_\rho )=\min _{\varvec{\theta }\in \Theta } \ell (\varvec{\theta })+\rho P(\varvec{\Lambda })$.

#### Condition 3.1

$q(\varvec{x};\varvec{\theta })$ fulfills the following conditions:

- For each $\varvec{x}\in {\mathbb {R}}^p$, function $q(\varvec{x};\varvec{\theta })$ on $\Theta$ is continuous.
- There exists a ${\mathbb {P}}$-integrable function $g(\varvec{x})$ such that for all $\varvec{x}\in {\mathbb {R}}^p$ and for all $\varvec{\theta }\in \Theta$ $|q(\varvec{x};\varvec{\theta })|\le g(\varvec{x})$.

Since $\ell (\varvec{\theta })$ is the discrepancy function in Eq. (3), $q(\varvec{x};\varvec{\theta })$ becomes a logarithm of density function of normal distribution; in this case, Condition 3.1 is satisfied. The following proposition shows that the prenet estimator converges almost surely to a true parameter which minimizes the quartimin criterion when $\rho \rightarrow 0$ as $n\rightarrow \infty$.

#### Proposition 3

Assume that Condition 3.1 is satisfied. We denote by $\Theta _q^*$ a set of true solutions of the following quartimin problem.

$$\begin{aligned} \min _{\varvec{\theta }\in \Theta }P_{\mathrm {qmin}}(\varvec{\Lambda })\;\text { subject to }\; \ell _*(\varvec{\theta })=\min _{\varvec{\theta }\in \Theta }\ell _*(\varvec{\theta }). \end{aligned}$$

Let $\rho _n$ ($n = 1,2,\dots$) be a sequence that satisfies $\rho _n > 0$ and $\lim _{n \rightarrow \infty }\rho _n = 0$. Let the prenet solution with $\gamma \rightarrow 0$ and $\rho = \rho _n$ be $\hat{\varvec{\theta }}_{\rho _n}$. Then we obtain

$$\begin{aligned} \lim _{n \rightarrow \infty }d(\hat{\varvec{\theta }}_{\rho _n},\Theta _q^*) = 0\quad a.s., \end{aligned}$$

where $$d({a},B)=\inf _{{b}\in B}d({a},{b})$$.

#### Proof

The proof is given in Appendix A.2. $\square$

#### Remark 3.1

Proposition 3 uses a set of true solutions $\Theta _q^*$ instead of one true solution $\varvec{\theta }_q^*$. This is because even if the quartimin solution does not have a rotational indeterminacy, it still has an indeterminacy with respect to sign and permutation of columns of the loading matrix.

#### Remark 3.2

The Geomin criterion (Yates, 1987) often produces a loading matrix similar to that obtained by the Quartimin criterion (Asparouhov and Muthén 2009). For the Geomin criterion, we add a small number to the loadings to address the identifiability problem (Hattori et al., 2017). Meanwhile, the prenet does not suffer from such a problem. The detailed discussion is described in Section S3 in the supplemental material.

## Algorithm

It is well known that the solutions estimated by the lasso-type penalization methods are not usually expressed in a closed form because the penalty term includes an indifferentiable function. As the objective function of the prenet is nonconvex, it is not easy to construct an efficient algorithm to obtain a global minimum. Here, we use the generalized EM algorithm, in which the latent factors are considered to be missing values. The complete-data log-likelihood function is increased with the use of the coordinate descent algorithm (Friedman et al., 2010), which is commonly used in the lasso-type penalization. Although our proposed algorithm is not guaranteed to attain the global minimum, our algorithm decreases the objective function at each step. The update equation of the algorithm and its complexity are presented in Appendix B.

### Efficient Algorithm for Sufficiently Large $\rho$

The prenet tends to be multimodal for large $\rho$ as is the case with the *k*-means algorithm. Therefore, we prepare many initial values, estimate the solutions for each initial value, and select a solution that minimizes the penalized loss function. In this case, it seems that we require heavy computational loads. However, we can construct an efficient algorithm for a sufficiently large $\rho$.

For sufficiently large $\rho$, the *i*th column of loading matrix $\varvec{\Lambda }$ has at most one nonzero element, denoted by $\lambda _{ij}$. With the EM algorithm, we can easily find the location of the nonzero parameter when the current value of the parameter is given. Assume that the (*i*, *j*)th element of the loading matrix is nonzero and the (*i*, *k*)th elements ($k \ne j$) are zero. Because the penalty function attains zero for sufficiently large $\rho$, it is sufficient to minimize the following function.

12 $$\begin{aligned} f(\lambda _{ij}) = \varvec{\lambda }_i^T \varvec{A}\varvec{\lambda }_i - 2\varvec{\lambda }_i^T\varvec{b}_i = a_{jj}\lambda _{ij}^2 - 2\lambda _{ij}b_{ij}. \end{aligned}$$

The minimizer is easily obtained by

13 $$\begin{aligned} {\hat{\lambda }}_{ij} = {b_{ij}}/{a_{jj}}. \end{aligned}$$

Substituting (13) into (12) gives us $f({\hat{\lambda }}_{ij}) = -\frac{b_{ij}^2}{a_{jj}}$. Therefore, the index *j* that minimizes the function $f(\lambda _{ij})$ is

$$\begin{aligned} j = \mathrm{argmax}_k\frac{b_{ik}^2}{a_{kk}}, \end{aligned}$$

and ${\lambda }_i$ is updated as ${\hat{\lambda }}_{ij} = {b_{ij}}/{a_{jj}}$ and ${\hat{\lambda }}_{ik} = 0$ for any *k* $\ne$ *j*.

### Selection of the Maximum Value of $\rho$

The value of $\rho _{\max }$, which is the minimum value of $\rho$ that produces the perfect simple structure, is easily obtained using $\hat{\varvec{\Lambda }}$ given by (13). Assume that ${\hat{\lambda }}_{ij}\ne 0$ and ${\hat{\lambda }}_{ik} = 0$ ($k \ne j$). Using the update equation of $\lambda _{ik}$ in (A.10) and the soft thresholding function in (A.12) of Appendix A, we show that the regularization parameter $\rho$ must satisfy the following inequality to ensure that $\lambda _{ik}$ is estimated to be zero.

$$\begin{aligned} \left| \frac{b_{ik}- a_{kj}{\hat{\lambda }}_{ij}}{a_{kk}+\rho \psi _i(1-\gamma ){\hat{\lambda }}_{ij}^2 }\right| \le \frac{\psi _i}{a_{kk}+\rho \psi _i(1-\gamma ){\hat{\lambda }}_{ij}^2 }\rho \gamma |{\hat{\lambda }}_{ij}|. \end{aligned}$$

Thus, the value of $\rho _{\max }$ is

$$\begin{aligned} \rho _{\max } = \max _{i} \max _{k \in C_i}\frac{|b_{ik}- a_{kj}{\hat{\lambda }}_{ij}|}{\gamma \psi _i|{\hat{\lambda }}_{ij}|}, \end{aligned}$$

where $$C_i = \{k|k \ne j, {\hat{\lambda }}_{ij} \ne 0\}$$.

### Estimation of the Entire Path of Solutions

The entire path of solutions can be produced with the grid of increasing values $\{\rho _1,\dots ,\rho _K\}$. Here, $\rho _K$ is (5.2), and $\rho _{1} = \rho _{K} \Delta \sqrt{\gamma }$, where $\Delta$ is a small value such as 0.001. The term $\sqrt{\gamma }$ allows us to estimate a variety of models even if $\gamma$ is small.

The entire solution path can be made using a decreasing sequence $\{\rho _K\dots ,\rho _1\}$, starting with $\rho _K$. The proposed algorithm at $\rho _K$ does not always converge to the global minimum, so that we prepare many initial values, estimate solutions for each initial value with the use of the efficient algorithm described in Sect. 4.1, and select a solution that minimizes the penalized log-likelihood function. We can use the warm start defined as follows: the solution at $\rho _{k-1}$ is computed using the solution at $\rho _k$. The warm start leads to improved and smoother objective value surfaces (Mazumder et al., 2011).

One may use the warm start with increasing $\rho$; that is, the solution with $\rho =0$ is obtained by the rotation technique with MLE, and then we gradually increase $\rho$, using the solution from the previous step. However, the decreasing sequence of $\rho$ has a significant advantage over the increasing sequence; the decreasing sequence allows the application to the $n<p$ case. With an increasing order, the solution with $\rho =0$ (MLE) is not available, and then the entire solution cannot be produced. Therefore, we adopt the warm start with decreasing sequence of $\rho$ instead of an increasing sequence.

Another method to estimate the entire solution is to use the cold start with multiple random starts. Although the cold start does not always produce a smooth estimate as a function of $\rho$, it can sometimes find a better solution than the warm start when the loss function has multiple local minima. However, the cold start often requires heavier computational loads than the warm start.

When $\rho$ is extremely small, the loss function becomes nearly flat due to rotational indeterminacy. However, in our experience, our proposed algorithm generally produces a smooth and stable estimate when the warm start is adopted. Even when the cold start is used, the estimate can often be stable for large sample sizes when $\rho$ is not extremely but sufficiently small, such as $\rho =10^{-4}$. However, when $n<p$, the maximum likelihood estimate cannot be obtained; therefore, the cold start often produces an unstable estimate with small $\rho$.

### Selection of the Regularization Parameter $\rho$

The estimate of the loading matrix depends on the regularization parameter $\rho$. As described in the Introduction, this study focus on two different purposes of the analysis: (i) exploratory factor analysis and (ii) clustering of variables. When the purpose of the analysis is (ii), we simply set $\rho \rightarrow \infty$ to achieve the perfect simple structure estimation. When the purpose of the analysis is (i), $\rho$ is selected by the AIC or the BIC (Zou et al., 2007);

$$\begin{aligned} \text {AIC}= & {} -2 n\ell (\hat{\varvec{\theta }}) + 2p_0,\\ \text {BIC}= & {} -2 n \ell (\hat{\varvec{\theta }}) +p_0 \log n, \end{aligned}$$

where $p_0$ is the number of nonzero parameters.

Our algorithm sometimes produces a loading matrix some of whose columns are zero vectors. In this case, the number of factors may be smaller than expected. The selection of the number of factors via the regularization is achieved by taking advantage of the zero column vectors estimation (Caner and Han, 2014; Hirose and Yamamoto, 2015).

## Monte Carlo Simulations

The performance of our proposed method is investigated through Monte Carlo simulations. The prenet penalization has two different purposes of analysis: clustering of variables and exploratory factor analysis. In this section, we investigate the performance in terms of both purposes. The comparison of various exploratory factor analysis methods is described in Sect. 5.2, and the investigation of clustering of variables is presented in Sect. 5.3.

### Simulation Models

In this simulation study, we use three simulation models as below.

**Model (A):**

$$\begin{aligned} \varvec{\Lambda }= & {} \left( \begin{array}{r@{\quad }r@{\quad }r@{\quad }r@{\quad }r@{\quad }r} 0.8&{}\quad 0 &{}\quad 0 &{}\quad 0 \\ 0 &{}\quad 0.7&{}\quad 0 &{}\quad 0 \\ 0 &{}\quad 0 &{}\quad 0.6&{}\quad 0 \\ 0 &{}\quad 0 &{}\quad 0 &{}\quad 0.5\\ \end{array} \right) \otimes \varvec{1}_{25}, \end{aligned}$$

where $\varvec{1}_{25}$ is a 25-dimensional vector with each element being 1.

**Model (B):**

The size of the loading matrix of Model (B) is the same as that of Model (A), and the nonzero factor loadings share the same values. However, all zero elements in Model (A) are replaced by small random numbers from $U(-0.3,0.3)$.

**Model (C):**

$$\begin{aligned} \varvec{\Lambda }= & {} (\varvec{\Lambda }_1^T, \ \varvec{\Lambda }_2^T)^T,\\ \varvec{\Lambda }_1= & {} \left( \begin{array}{rrrrrrrrrrrrrr} 0.79 &{} 0.00 &{} 0.00 &{} 0.49 &{} 0.50 &{} 0.00 &{} 0.68 &{} 0.29 &{} 0.66 &{} 0.33 &{} 0.00 &{} 0.00 &{} 0.62 \\ 0.00 &{} 0.77 &{} 0.00 &{} 0.53 &{} 0.00 &{} 0.50 &{} 0.32 &{} 0.66 &{} 0.00 &{} 0.00 &{} 0.62 &{} 0.34 &{} -0.64\\ 0.00 &{} 0.00 &{} 0.76 &{} 0.00 &{} 0.52 &{} 0.48 &{} 0.00 &{} 0.00 &{} 0.34 &{} 0.66 &{} 0.33 &{} 0.62 &{} 0.00\\ \end{array} \right) ^T,\\ \varvec{\Lambda }_2= & {} \left( \begin{array}{rrrrrrrrrrrrrr} -0.62 &{} 0.62 &{} -0.62 &{} 0.00 &{} 0.00 &{} 0.43 &{} 0.47 &{} 0.00 &{} 0.42 &{} 0.43 &{} 0.00 &{} 0.36 &{} 0.26 \\ 0.64 &{} 0.00 &{} 0.00 &{} 0.67 &{} -0.67 &{} 0.58 &{} 0.00 &{} 0.50 &{} 0.57 &{} 0.00 &{} 0.50 &{} 0.38 &{} 0.43 \\ 0.00 &{} -0.61 &{} 0.61 &{} -0.63 &{} 0.63 &{} 0.00 &{} 0.57 &{} 0.48 &{} 0.00 &{} 0.57 &{} 0.46 &{} 0.38 &{} 0.38 \\ \end{array} \right) ^T. \end{aligned}$$

The simulation is conducted for both orthogonal and oblique models on (A) and an orthogonal model on (B) – (C). For Model (A), we write the orthogonal and oblique models as “Model (A-ORT)” and “Model (A-OBL),” respectively. Here, “ORT” and “OBL” are abbreviations for “orthogonal” and “oblique,” respectively. The factor correlations for the oblique model are set to be 0.4. The unique variances are calculated by $\varvec{\Psi } = \mathrm{diag} (\varvec{I}_p - \varvec{\Lambda }\varvec{\Phi }\varvec{\Lambda }^T)$.

In Model (A), the loading matrix possesses the perfect simple structure. In such cases, the prenet is expected to perform well because it is able to estimate the perfect simple structure for large $\rho$ (Proposition 1). Note that $p=100$ on Model (A); therefore, maximum likelihood estimate cannot be available when $n < 100$.

The loading matrix of Model (B) is close to but not exactly a perfect simple structure. In this case, the prenet is expected to perform well when both $\rho$ and $\gamma$ are close to zero, thanks to Proposition 3. Meanwhile, the lasso would not perform well; a small illustrative example with intuitive description is presented in Section S1 in supplemental material.

In Model (C), the loading matrix is sparse but more complex than the perfect simple structure. The loading matrix is a rotated centroid solution of the Thurstone’s box problem, reported in Thurstone (1947). We use data(ThurstoneBox26) in the fungible package in R to obtain the loading matrix. With the original loading matrix, some of the unique variances can be larger than 1 with $\varvec{\Psi } = \mathrm{diag} (\varvec{I}_p - \varvec{\Lambda }\varvec{\Lambda }^T)$; therefore, the elements of the original loading matrix are multiplied by 0.83. Furthermore, to enhance the sparsity, factor loadings whose absolute values are less than 0.1 are replaced by 0.

### Accuracy Investigation

The model parameter is estimated by the prenet penalty with $\gamma = 1.0$, 0.1, 0.01, the lasso, the MCP (Zhang, 2010)

$$\begin{aligned} \rho P(\varvec{\Lambda };\rho ;\gamma )= & {} \sum _{i = 1}^p\sum _{j = 1}^m\rho \int _0^{|\lambda _{ij}|}\left( 1-\frac{x}{\rho \gamma }\right) _+dx \end{aligned}$$

with $\gamma = 3$, and the elastic net with $\gamma =0.1$. The regularization parameter $\rho$ is selected by the AIC and the BIC. We also compute a limit, $\displaystyle \lim _{\rho \rightarrow +0}{\hat{\Lambda }}_{\rho }$, where ${\hat{\Lambda }}_{\rho }$ is the estimate of the loading matrix obtained with a regularization parameter $\rho$. We note that $\displaystyle \lim _{\rho \rightarrow +0}{\hat{\Lambda }}_{\rho }$ corresponds to the factor rotation with MLE (Hirose and Yamamoto, 2015). In particular, the estimate with $\rho \rightarrow +0$ and $\gamma \rightarrow +0$ is equivalent to that obtained by the quartimin rotation with MLE, thanks to Proposition 3. We also conduct rotation techniques with MLE: varimax rotation (Kaiser, 1958) for the orthogonal model and promax rotation (Hendrickson and White, 1964) for the oblique model. When MLE cannot be found due to $n<p$, we conduct the lasso and obtain the approximation of the MLE with $\rho \rightarrow +0$.

The warm start is used for Models (A) and (B). The dimension of these models is $p=100$, and then the warm start is stabler than the cold start for small $\rho$ in our experience. Meanwhile, we adopt the cold start on Model (C) because Thurstone’s box problem tends to possess multiple local minima.

In our implementation, the estimate of the elastic net sometimes diverges for the oblique model. Scharf and Nestler (2019a) reported the same phenomenon. To address this issue, we add a penalty $\zeta \log |\varvec{\Phi }|$ to Eq. (2) with $\zeta =0.01$. This penalty is based on Lee (1981), a conjugate prior for Wishart distribution from a Bayesian viewpoint. We remark that the prenet does not tend to suffer from this divergence issue even if $\zeta =0$ in our experience. This is probably because the prenet does not shrink all loadings to zero, thanks to Proposition 1. For example, assume that ${\hat{\sigma }}_{ij} = {\hat{\lambda }}_{im}{\hat{\lambda }}_{jk} {\hat{\phi }}_{km}$ ($k \ne m$). When the elastic net penalization is adopted, both ${\hat{\lambda }}_{im}$ and ${\hat{\lambda }}_{jk}$ are close to zero with a large $\rho$. When $s_{ij}$ is large, ${\hat{\phi }}_{km}$ must be large to get $\sigma _{ij} \approx s_{ij}$. Accordingly, the value of ${\hat{\phi }}_{km}$ can be significantly large; it can be greater than 1. Meanwhile, the prenet may not suffer from this problem because either ${\hat{\lambda }}_{im}$ and ${\hat{\lambda }}_{jk}$ can become large.

For each model, $T = 1000$ data sets are generated with $N(\varvec{0},\varvec{\Lambda }\varvec{\Phi }\varvec{\Lambda }^T + \varvec{\Psi })$. The number of observations is $n =50, 100$, and 500. To investigate the performance of various penalization procedures, we compare the root mean squared error (RMSE) over $T=1000$ simulations, which is defined by

$$\begin{aligned} \text {RMSE} = \frac{1}{T} \left( \sum _{s = 1}^{T} \frac{\Vert \varvec{\Lambda } - \hat{\varvec{\Lambda }}^{(s)} \Vert _{F}^2}{pm} \right) ^{1/2}, \end{aligned}$$

where $\hat{ \varvec{\Lambda }}^{(s)}$ is the estimate of the loading matrix using the *s*th dataset. We also compare the rate of nonzero factor loadings for Models (A) and (B). Because the loading matrix is not identifiable due to permutation and sign of columns, we change the permutation and sign such that RMSE is minimized. It is not fair to compare the rate of nonzero loadings for $\rho \rightarrow +0$, because the estimated loading matrix cannot become sparse. Thus, we apply the hard-thresholding with a cutoff value being 0.1, the default of the loadings class in R.

**Fig. 2:**
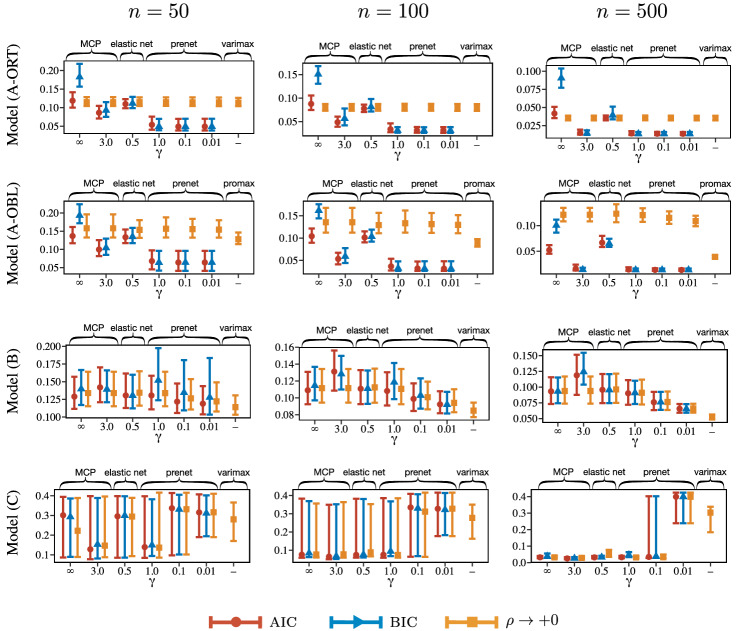
RMSE of factor loadings. The upper and lower bars represent 95th and 5th percentiles, respectively. Here, “$\rho \rightarrow +0$” denotes a limit of the estimate of the factor loadings, $\displaystyle \lim _{\rho \rightarrow +0}{\hat{\Lambda }}_{\rho }$, which corresponds to the factor rotation.

**Fig. 3:**
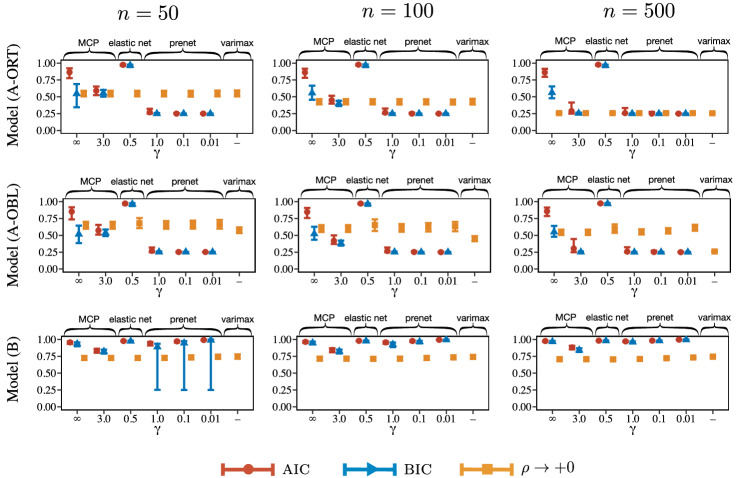
Rate of nonzero factor loadings. The upper and lower bars represent 95th and 5th percentiles, respectively. Here, “$\rho \rightarrow +0$” denotes a limit of the estimate of the factor loadings, $\displaystyle \lim _{\rho \rightarrow +0}{\hat{\Lambda }}_{\rho }$, which corresponds to the factor rotation.

The results for RMSE and the rate of nonzero factor loadings are depicted in Figs. 2 and 3, respectively. For reference, true positive rate (TPR) and false positive rate (FPR) of the loading matrix are depicted in Figures S1.4 and S1.5 in the supplemental material. The range of the error bar indicates 90% confidence interval; we calculate 5% and 95% quantiles over 1000 simulation results and use them as the boundaries of the error bar. We obtain the following empirical observations from these figures.

**Model (A-ORT):** The prenet penalization outperforms the existing methods in terms of RMSE when the regularization parameter is selected by the model selection criteria. It is also seen that the performance of the prenet is almost independent of $\gamma$. When $\rho \rightarrow +0$, all of the estimation procedures yield similar performances. When the $\rho$ is selected by the model selection criteria, the rate of nonzero loadings of the prenet is almost 0.25, that is the true rate. Considering the TPR result in Figure S1.4 in the supplemental material, the prenet with AIC or BIC correctly estimates the true zero/nonzero pattern. Meanwhile, the MCP, elastic net, and lasso tend to select a denser model than the true one.
**Model (A-OBL):** The result for the oblique model is similar to that for the orthogonal model, but the oblique model tends to produce larger RMSEs than the orthogonal model for most cases. The $\rho \rightarrow +0$ produces larger RMSE than that with the regularization parameter $\rho$ selected by model selection criteria. This is probably because the loss function becomes flat as $\rho \rightarrow +0$. Therefore, the regularization may help improve the accuracy. We note that the promax rotation, which corresponds to $\rho \rightarrow +0$, turns out to be stable.
**Model (B):** When *n* is large, the prenet with small $\gamma$ and varimax rotation produces small RMSEs. Because the true values of cross-loadings (small loadings) are close to but not exactly zero, the $L_1$ type regularization that induces a sparse loading matrix does not work well. The prenet with $\gamma =0.01$ achieves the sparse estimation but produces a loading matrix that is similar to the quartimin rotation, resulting in a nonsparse loading matrix. We also observe that the prenet with BIC sometimes results in too sparse loading matrix when $n=50$.
**Model (C):** For small *n*, all methods result in large RMSE. For large *n*, the $L_1$ regularization methods, including the lasso, MCP, elastic net, and prenet with large $\gamma$ yield small RMSE. However, the prenet with small $\gamma$ and varimax rotation, which tend to estimate non-sparse loading matrix, produce large RMSE. Indeed, the average value of loading matrix in the supplemental material shows that the prenet with small $\gamma$ is biased. Furthermore, the varimax rotation with true loading matrix does not approximate the true one. Therefore, when the loading matrix is sparse but does not have the perfect simple structure, the lasso-type penalization or prenet with $\gamma =1$ would perform well.

### Investigation of Clustering via Perfect Simple Structure Estimation

**Fig. 4:**
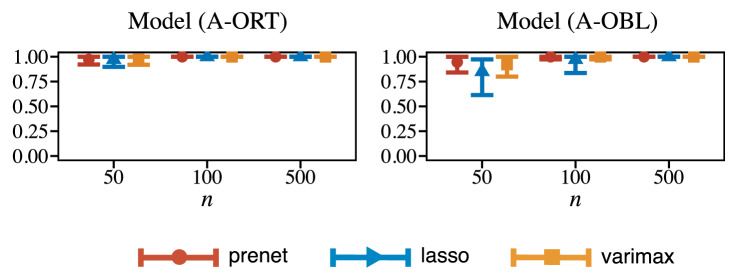
Adjusted Rand Index (ARI) of the clustering results.

As shown in Proposition 1, our proposed method allows the clustering of variables via the perfect simple structure estimation. We investigate the clustering accuracy on Model (A); the true loading matrix has the perfect simple structure, and then we know the true clusters. Figure 4 shows the Adjusted Rand Index (ARI) between true clusters and those obtained by prenet, lasso, and varimax. The range of the error bar indicates 90% confidence interval; we calculate 5% and 95% quantiles over 1000 simulation results and use them as the boundaries of the error bar.

The clustering via prenet is achieved by perfect simple structure estimation. The lasso and varimax cannot always estimate the perfect simple structure. Therefore, we estimate the clusters as follows: for *i*th row vector of $\hat{\varvec{\Lambda }}$, say $\hat{\varvec{\lambda }}_i = ({\hat{\lambda }}_{i1}, \dots , {\hat{\lambda }}_{im})^T$, the *i*th variable belongs to *j*th cluster, where $j = \mathop {\mathrm{arg~max}}\limits _{j \in \{1,\dots ,m\}}(|{\hat{\lambda }}_{ij}|)$. The regularization parameter for the lasso is $\rho \rightarrow +0$, which corresponds to a special case of the component loss criterion (Jennrich, 2004, 2006) with MLE.

The result of Fig. 4 shows that the prenet and varimax result in almost identical ARIs and are slightly better than the lasso when $n=50$ on Model (A-ORT). All methods correctly detect the true clusters when $n=100$ and $n=500$. For Model (A-OBL), the prenet performs slightly better than the varimax when $n=50$. As with the orthogonal model, the prenet and varimax correctly detect the true clusters when $n=100$ and $n=500$. The lasso performs worse than the other two methods for small sample sizes, suggesting that the prenet or varimax would be better if the clustering of variables is the purpose of the analysis.

## Analysis of Big Five Personality Traits

We apply the prenet penalization to the survey data regarding the big five personality traits collected from Open Source Psychometrics Project (https://openpsychometrics.org/). Other real data applications (electricity demand and fMRI data) are described in Section S2 of the supplemental material. $n=8582$ responders in the US region are asked to assess their own personality based on 50 questions developed by Goldberg (1992). Each question asks how well it describes the statement of the responders on a scale of 1–5. It is well known that the personality is characterized by five common factors; therefore, we choose $m=5$. Several earlier researchers showed that the loading matrix may not possess the perfect simple structure due to the small cross-loadings (Marsh et al., 2010, 2013; Booth and Hughes, 2014); therefore, we do not aim at estimating the perfect simple structure with $\rho \rightarrow \infty$ in this analysis. We first interpret the estimated model and then investigate the performance of the prenet penalization with $\rho$ selected by model selection criteria for various sample sizes. The impact of the regularization parameter on the accuracy is also studied.

### Interpretation of Latent Factors

**Fig. 5:**
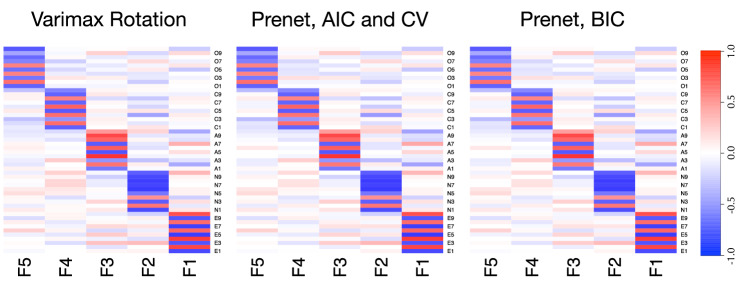
Heatmaps of the loading matrices on big five personality traits data. Each cell corresponds to the factor loading, and the depth of color indicates the magnitude of the value of the factor loading.

We first apply the prenet penalization and the varimax rotation with maximum likelihood estimate and compare the loading matrices estimated by these two methods. With the prenet penalization, we choose a regularization parameter using AIC, BIC, and tenfold cross-validation (CV) with $\gamma =1$. The regularization parameter selected by the AIC and CV is $\rho = 7.4 \times 10^{-4}$, and that selected by the BIC is $\rho = 2.9\times 10^{-3}$. The heatmaps of the loading matrices are shown in Fig. 5. The result of Fig. 5 shows that these heatmaps are almost identical; all methods are able to detect the five personality traits appropriately. We also observe that the result is almost independent of $\gamma$. These similar results may be due to the large sample sizes.

We explore the estimates of cross-loadings whose absolute values are larger than 0.3; it would be reasonable to regard that these cross-loadings affect the items. There exists four items that include such large absolute values of cross-loadings, and factor loadings related to these four items are shown in Table 1.

**Table 1:** Factor loadings of four items estimated by the prenet penalization with $\gamma =0.01$. The regularization parameter, $\rho$, is selected by the BIC. The cross-loadings whose absolute values are larger than 0.3 are written in bold.

Item	F1	F2	F3	F4	F5
A2: Am not interested in other people’s problems.	**0**.**341**	$-$0.062	$-$0.525	$-$0.020	0.070
A7: Have a soft heart.	-**0**.**317**	0.089	0.615	0.008	$-$0.010
A10: Do not have a good imagination.	**0**.**347**	$-$0.164	$-$0.375	0.116	0.082
C4: Change my mood a lot.	$-$0.083	**0**.**365**	0.033	$-$0.548	0.022

The loading matrix is estimated by the prenet penalization with $\gamma =0.01$. The regularization parameter $\rho$ is selected by the BIC. The five factors represent “F1: extraversion,” “F2: neuroticism,” “F3: agreeableness,” “F4: conscientiousness,” and “F5: openness to experience.” For reference, the complete loading matrix is shown in Tables S2.5 and S2.6 of the supplemental material.

The three items, A2, A7, and A10, are affected by “F1: extraversion” and “F3: agreeableness.” The main and cross-loadings on the same item have opposite signs. We may make a proper interpretation of the factor loadings. For example, as for the question “A7: Have a soft heart,” it is easy to imagine some people who have an extraversion cannot be kind. They are interested in a profit from a person rather than the situation that the person is in now; thus, they can become selfish to get the profit even if the person’s feelings are hurt. Booth and Hughes (2014) also reported similar results of such cross-loadings. They mentioned that these cross-loadings were due to the overlap in content between extraversion and agreeableness.

Furthermore, we perform $M=100$ subsampling simulation with $n=500$ to investigate whether these cross-loadings can be found with small sample sizes. We compare the performance of four estimation methods: the prenet, lasso, MCP, and varimax rotation. For regularization methods, $\rho$ is selected by the BIC. We set $\gamma =1$ and $\gamma =0.01$ for the prenet and $\gamma =3$ for MCP.

**Table 2:** The number of times that the absolute values of four cross-loadings exceed 0.3. For regularization methods, $\rho$ is selected by the BIC.

	Prenet ($\gamma =1$)	Prenet ($\gamma =0.01$)	Lasso	MCP	Varimax
A2	25	85	35	69	81
A7	10	72	18	45	61
A10	29	90	40	68	80
C4	20	94	49	78	94

Table 2 shows the number of times that the absolute values of these four cross-loadings exceed 0.3. The results show that the prenet with $\gamma =0.01$ most frequently identifies these four cross-loadings among $M=100$ simulations.

### RMSE Comparison

We investigate the performance of the prenet in terms of estimation accuracy of the loading matrix through subsampling simulation. First, the dataset is randomly split into two datasets, $\varvec{X}_1$ and $\varvec{X}_2$, without replacement. The sample sizes of $\varvec{X}_1$ and $\varvec{X}_2$ are $n/2 = 4291$. The $\varvec{X}_1$ is used for estimating a loading matrix with large sample sizes; we perform the varimax rotation with MLE and regard the estimated loading matrix as a true loading matrix, say $\varvec{\Lambda }_{\mathrm{true}}$. The true loading matrix is almost identical to the loading matrix obtained by the varimax with the entire dataset. We remark that the true loading matrix is also similar to the Model (B) of the Monte Carlo simulation described in Sect. 5.1.

**Fig. 6:**
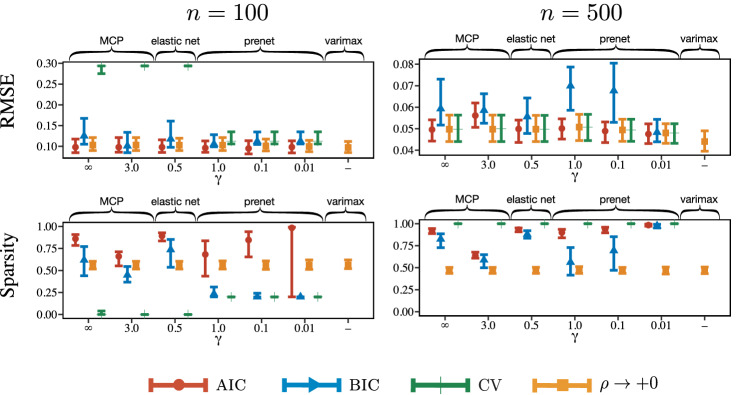
RMSE and rate of nonzero loadings when $n=100$ and 500. Here, “$\rho \rightarrow +0$” denotes a limit of the estimate of the factor loadings, $\displaystyle \lim _{\rho \rightarrow +0}{\hat{\Lambda }}_{\rho }$, which corresponds to the factor rotation.

The performance is investigated by subsampling the observations from $\varvec{X}_2$ with $n=100$ and $n=500$. Figure 6 depicts RMSE and rate of nonzero loadings for *n* random subsampled data over 100 simulations. The RMSE is defined as

$$\begin{aligned} \text {RMSE} = \frac{1}{100} \left( \sum _{s = 1}^{100} \frac{\Vert \varvec{\Lambda }_{\mathrm{true}} - \hat{\varvec{\Lambda }}^{(s)} \Vert _{F}^2}{pm} \right) ^{1/2}, \end{aligned}$$

where $\hat{ \varvec{\Lambda }}^{(s)}$ is the estimate of the loading matrix using the *s*th subsampled data. We apply the lasso, MCP with $\gamma =3$, prenet with $\gamma =1$, 0.1, 0.01, and the varimax rotation with MLE. The regularization parameter $\rho$ is selected by the AIC, BIC, and tenfold CV. We also compute the loading matrix when $\rho \rightarrow +0$, which results in the solution of the factor rotation with MLE. The nonzero pattern of the loading matrix for $\rho \rightarrow +0$ is estimated by a hard-thresholdings with a cutoff value being 0.1. The range of the error bar indicates 90% confidence interval over 100 simulations.

We have the following empirical observations from Fig. 6:

- The smaller the number of observations is, the sparser the solution is. An earlier study has shown that the model selection criterion can select a parsimonious model with small sample sizes in general frameworks (Cudeck and Henly, 1991).
- The BIC results in larger RMSE and lower rate of nonzero loadings than other criteria, especially for small sample sizes. Therefore, the BIC tends to select sparse solutions, and some of the small nonzero factor loadings are estimated to be zero.
- When the lasso or MCP is applied, the CV results in poor RMSE when $n=100$. This is because the estimated loading matrix is too sparse; it becomes (almost) zero matrix. When the prenet is applied, such a loading matrix cannot be obtained thanks to Proposition 1.
- With the prenet, small $\gamma$ tends to estimate a dense loading matrix and produce good RMSE. A similar tendency is found in Model (B) of the Monte Carlo simulation, described in Sect. 5.2.

### Impact of Tuning Parameters

**Fig. 7:**
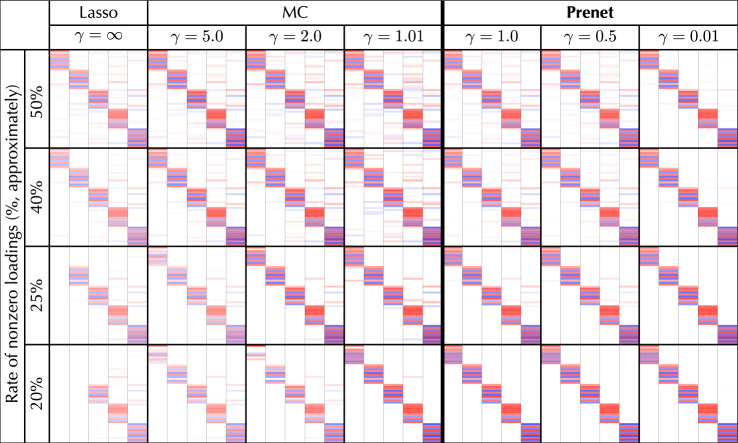
Heatmaps of the loading matrices on big five personality traits data for various values of tuning parameters on the MCP and the prenet penalization.

We investigate the impact of the tuning parameters $(\rho , \gamma )$ on the estimation of the loading matrix. Figure 7 depicts the heatmaps of the loading matrices for various values of tuning parameters on the MCP and the prenet penalization. We find the tuning parameters so that the degrees of sparseness (proportion of nonzero values) of the loading matrix are approximately 20%, 25%, 40%, and 50%. For the MCP, we set $\gamma = \infty$ (i.e., the lasso), 5.0, 2.0, and 1.01. For prenet penalty, the values of gamma are $\gamma =1.0,$ 0.5,  and 0.01. Each cell describes the elements of the factor loadings as with Fig. 5.

From Fig. 7, we obtain the empirical observations as follows.

- With the prenet penalization, the characteristic of five personality traits are appropriately extracted for any values of tuning parameters, which suggests that the prenet penalization is relatively robust against the tuning parameters.
- The prenet penalization is able to estimate the perfect simple structure when the degree of sparseness is 20%. On the other hand, with the MCP, we are not able to estimate the perfect simple structure even when $\gamma$ is sufficiently small.
- With the lasso, the number of factors becomes less than five when the degrees of sparsity are 20% and 25%; the five personality traits are not able to be found. When the value of $\gamma$ is not sufficiently large, the MCP produces five factor model.

## Discussion

We proposed a prenet penalty, which is based on the product of a pair of parameters in each row of the loading matrix. The prenet aims at the estimation of not only sparsity but also the simplicity. Indeed, the prenet is a generalization of the quartimin criterion, one of the most popular oblique techniques for simple structure estimation. Furthermore, the prenet is able to estimate the perfect simple structure, which gave us a new variables clustering method using factor models. The clustering of variables opens the door to the application of the factor analysis to a wide variety of sciences, such as image analysis, neuroscience, marketing, and biosciences.

The prenet penalization has two different purposes of analysis: clustering of variables and exploratory factor analysis. The way of using the prenet penalization depends on the purpose of the analysis. When the purpose of the analysis is the clustering of variables, the regularization parameter is set to be $\rho \rightarrow \infty$ to achieve the perfect simple structure estimation. It is shown that the prenet performs better than the lasso and varimax in terms of clustering accuracy, as described in Sect. 5.3. Furthermore, the real data analyses in Section S2 in the supplemental material show the superiority of the prenet over the conventional clustering methods, such as the *k*-means clustering. When the purpose of the analysis is exploratory factor analysis, the perfect simple structure estimation is not necessarily needed. In this case, the regularization parameter is selected by the model selection criteria. The numerical results show that the prenet penalization performs well when an appropriate value of $\gamma$ is selected.

Over a span of several decades, a number of researchers have developed methods for finding a simple loading matrix (e.g., Kaiser, 1958; Hendrickson and White, 1964) in the Thurstonian sense (Thurstone, 1947). As the simple structure is a special case of sparsity, it seems the lasso-type sparse estimation is more flexible than the prenet. Indeed, the recent trend in the exploratory factor analysis literature is to find a loading matrix that possesses the sparsity rather than simplicity (Jennrich, 2004, 2006; Trendafilov, 2013; Scharf and Nestler, 2019b, a).

Nevertheless, the lasso-type sparse estimation is not as flexible as expected. As mentioned in Model (B) in Monte Carlo simulation and Section S1 in the supplemental material, the lasso cannot often approximate the true loading matrix when the cross-loadings are not exactly but close to zero. Because some factor loadings are estimated to be *exactly* zero with the lasso, some other factor loadings turn out to be excessively large, which causes the difficulty in interpretation.

For this reason, we believe *both sparsity and simplicity* play important roles in the interpretation. The sparse estimation automatically produces the nonzero pattern of the loading matrix, which allows us to interpret the latent factors easily. In addition, simplicity is also helpful for the interpretation, as shown in Thurstone (1947). The prenet penalization is able to achieve simplicity and sparsity simultaneously. Indeed, a sparse loading matrix is estimated thanks to the penalty based on the absolute term in $\sum _{i,j,k}|\lambda _{ij}\lambda _{ik}|$. In addition, simplicity is also achieved because it generalizes the quartimin criterion that often produces a simple structure (Jennrich and Sampson, 1966). Furthermore, with a large value of the regularization parameter, the loading matrix enjoys the perfect simple structure. Meanwhile, the existing methods cannot always produce a loading matrix that is both sparse and simple. For example, the lasso produces a loading matrix that is sparse but not always simple.

The structural equation modeling (SEM) has been widely used in the social and behavioral sciences. The SEM covers a wide variety of statistical models, including the factor analysis model and the regression model. An analyst develops an assumption of causal relationship and determines whether the assumption is correct or not by testing the hypothesis or evaluating the goodness of fit indices. Recently, several researchers have proposed regularized structural equation models (Jacobucci et al., 2016; Huang et al., 2017; Huang, 2018). The analyst set lasso-type penalties to specific model parameters to conduct an automatic selection of the causal relationship, enhancing the flexibility in model specification. The application of the prenet to the SEM would be an interesting future research topic.

The lasso-type regularization extracts only the nonzero pattern of parameters. In some cases, the analyst needs to detect not only the nonzero pattern of parameters but also a more complex parameter structure. The penalty must be determined depending on the structure of the parameter. For example, when the analyst needs to estimate either $\theta _1$ or $\theta _2$ to be zero, the prenet penalty would be more useful than the lasso. More generally, when one of $\theta _1,\dots ,\theta _k$ is exactly or close to zero, we may use the Geomin-type penalty, $\prod _{j=1}^k|\theta _j|$. An application of a penalty that leads to structured sparsity would further enhance the flexibility of the analysis but beyond the scope of this research. We would like to take this as a future research topic.

Another interesting extension to the prenet penalization is the clustering of not only variables but also observations. This method is referred to as biclustering (e.g., Tan and Witten, 2014; Flynn and Perry, 2020). To achieve this, we may need an entirely new formulation along with an algorithm to compute the optimal solution. This extension should also be a future research topic.

## Supplemental Materials

**Further numerical and theoretical investigations** Analyses of resting-state fMRI data, electricity demand data, a loading matrix of big five data, and comparison with Geomin criterion.

**R-package fanc** R-package fanc containing code that performs our proposed algorithm.

**Loadings** Average of the estimated loading matrices for Monte Carlo simulations in Sect. 5 with excel files.

### Supplementary Information

Below is the link to the electronic supplementary material.

Supplementary file 1 (pdf 1749 KB)

Supplementary file 2 (xlsx 114 KB)

Supplementary file 3 (xlsx 113 KB)

Supplementary file 4 (xlsx 111 KB)

Supplementary file 5 (xlsx 116 KB)

Supplementary file 6 (xlsx 113 KB)

Supplementary file 7 (xlsx 111 KB)

Supplementary file 8 (xlsx 126 KB)

Supplementary file 9 (xlsx 126 KB)

Supplementary file 10 (xlsx 125 KB)

Supplementary file 11 (xlsx 31 KB)

Supplementary file 12 (xlsx 31 KB)

Supplementary file 13 (xlsx 30 KB)

## Proofs

### Proof of Proposition 2

Because of Proposition 1, with the prenet, ${\hat{\lambda }}_{ij}{\hat{\lambda }}_{ik} = 0$ as $\rho \rightarrow \infty$. Thus, the prenet solution satisfies (9) as $\rho \rightarrow \infty$. We only need to show that the minimization problem of loss function $\ell _{\text {ML}}(\varvec{\Lambda },\varvec{\Psi })$ is equivalent to that of $\Vert \varvec{S} - \varvec{\Lambda }\varvec{\Lambda }^T\Vert _{F}^2$. The inverse covariance matrix of the observed variables is expressed as

$$\begin{aligned} \varvec{\Sigma }^{-1} = \varvec{\Psi }^{-1} - \varvec{\Psi }^{-1}\varvec{\Lambda }(\varvec{\Lambda }^T\varvec{\Psi }^{-1}\varvec{\Lambda } + \varvec{I}_m)^{-1}\varvec{\Lambda }^T\varvec{\Psi }^{-1}. \end{aligned}$$

Because $\varvec{\Lambda }^T\varvec{\Lambda } = \varvec{I}_m$, we obtain

$$\begin{aligned} \varvec{\Sigma }^{-1} = \alpha ^{-1}\varvec{I}_p - \frac{\alpha ^{-2}}{\alpha ^{-1} + 1}\varvec{\Lambda }\varvec{\Lambda }^T. \end{aligned}$$

The determinant of $\varvec{\Sigma }$ can be calculated as

$$\begin{aligned} |\varvec{\Sigma }| = \alpha ^{p-m}(1+\alpha )^m. \end{aligned}$$

Then, the discrepancy function in (3) is expressed as

$$\begin{aligned} \frac{1}{2}\left\{ \mathrm{tr}(\alpha ^{-1}\varvec{S}) - \frac{\alpha ^{-2}}{\alpha ^{-1} + 1}\mathrm{tr}\left( \varvec{\Lambda }^T\varvec{S}\varvec{\Lambda } \right) + p\log \alpha + m \log \left( 1+\frac{1}{\alpha } \right) - \log |\varvec{S}| -p \right\} . \end{aligned}$$

Because $\alpha$ is given and $\Vert \varvec{S} - \varvec{\Lambda }\varvec{\Lambda }^T\Vert _{F}^2 = -2\mathrm{tr} \left( \varvec{\Lambda }^T\varvec{S}\varvec{\Lambda } \right) + C.$ with constant value *C*, we can derive (10).

### Proof of Proposition 3

Recall that $\hat{\varvec{\theta }}$ is an unpenalized estimator that satisfies $\displaystyle {\ell }(\hat{\varvec{\theta }})=\min _{\varvec{\theta } \in \Theta } {\ell }(\varvec{\theta })$ and $\hat{\varvec{\theta }}_q$ is a quartimin solution obtained by the following problem.

$$\begin{aligned} \min _{\varvec{\theta } \in \Theta }P_{\mathrm{qmin}}(\varvec{\Lambda }), \text{ subject } \text{ to } \quad \ell (\varvec{\theta }) = \ell (\hat{\varvec{\theta }}). \end{aligned}$$

First, we show that

A.1 $$\begin{aligned} \lim _{n\rightarrow \infty } d(\hat{\varvec{\theta }}_q,\Theta _q^*)=0\;\;a.s. \end{aligned}$$

From the assumptions, as the same manner of Chapter 6 in Pfanzagl (1994), we can obtain the following strong consistency.

A.2 $$\begin{aligned} \lim _{n\rightarrow \infty } d(\hat{\varvec{\theta }},\Theta _*)=0\;\text { and }\;\lim _{n\rightarrow \infty } d(\hat{\varvec{\theta }}_{\rho _n},\Theta _*)=0\quad a.s., \end{aligned}$$

where $\Theta _*:=\{\varvec{\theta }\in \Theta \mid \ell _*(\varvec{\theta })=\min _{\varvec{\theta }\in \Theta }\ell _*(\varvec{\theta })\}$. When $\lim _{n\rightarrow \infty } d(\hat{\varvec{\theta }},\Theta _*)=0$, for all $\epsilon >0$, by taking *n* large enough, we have

$$\begin{aligned} \Vert \hat{\varvec{\Lambda }}-\varvec{\Lambda }_*\Vert _{F}<\epsilon \quad a.s. \end{aligned}$$

for some $(\mathrm{vec}(\varvec{\Lambda }_*)^T,\mathrm{diag}(\varvec{\Psi }_*)^T,\mathrm{vech}(\varvec{\Phi }_*)^T)^T\in \Theta _*$. From the uniform continuity of $P_{\mathrm {qmin}}$ on $\Theta$ and the fact that $\Vert \hat{\varvec{\Lambda }}\varvec{T}-\varvec{\Lambda }_*\varvec{T}\Vert _{F}=\Vert \hat{\varvec{\Lambda }}-\varvec{\Lambda }_*\Vert _{F}$ for any $\varvec{T}\in {\mathcal {O}}(m)$, we have

A.3 $$\begin{aligned} \sup _{\varvec{T}\in {\mathcal {O}}(m)} |P_{\mathrm {qmin}}(\hat{\varvec{\Lambda }}\varvec{T})-P_{\mathrm {qmin}}(\varvec{\Lambda }_*\varvec{T})|<\epsilon \quad a.s. \end{aligned}$$

Write $\hat{\varvec{T}}:=\mathop {\arg \min }_{\varvec{T}\in {\mathcal {O}}(m)}P_{\mathrm {qmin}}(\hat{\varvec{\Lambda }}\varvec{T})$ and $\varvec{T}_*:=\mathop {\arg \min }_{\varvec{T}\in {\mathcal {O}}(m)}P_{\mathrm {qmin}}(\varvec{\Lambda }_*\varvec{T})$. We have

$$\begin{aligned} P_{\mathrm {qmin}}(\hat{\varvec{\Lambda }}\hat{\varvec{T}})-P_{\mathrm {qmin}}(\varvec{\Lambda }_*\hat{\varvec{T}}) \le P_{\mathrm {qmin}}(\hat{\varvec{\Lambda }}\hat{\varvec{T}})-P_{\mathrm {qmin}}(\varvec{\Lambda }_*\varvec{T}_*) \le P_{\mathrm {qmin}}(\hat{\varvec{\Lambda }}\varvec{T}_*)-P_{\mathrm {qmin}}(\varvec{\Lambda }_*\varvec{T}_*). \end{aligned}$$

From this, it follows that

$$\begin{aligned} |P_{\mathrm {qmin}}(\hat{\varvec{\Lambda }}\hat{\varvec{T}})-P_{\mathrm {qmin}}(\varvec{\Lambda }_*\varvec{T}_*)| \le \sup _{\varvec{T}\in {\mathcal {O}}(m)} |P_{\mathrm {qmin}}(\hat{\varvec{\Lambda }}\varvec{T})-P_{\mathrm {qmin}}(\varvec{\Lambda }_*\varvec{T})|. \end{aligned}$$

Thus, using (A.3), we obtain (A.1).

Next, as the similar manner of Proposition 15.1 in Foucart and Rauhut (2013), we prove $\lim _{n\rightarrow \infty } d(\hat{\varvec{\theta }}_{\rho _n},\Theta _q^*)=0$, *a*.*s*. By the definition of $\hat{\varvec{\theta }}_{\rho _n}$, for any $\rho _n>0$ we have

A.4 $$\begin{aligned} \ell (\hat{\varvec{\theta }}_{{\rho _n}}) + \rho _n P_{\mathrm{qmin}}(\hat{\varvec{\Lambda }}_{{\rho _n}}) \le \ell (\hat{\varvec{\theta }}_q) +\rho _n P_{\mathrm{qmin}}(\hat{\varvec{\Lambda }}_q) \end{aligned}$$

and

A.5 $$\begin{aligned} \ell (\hat{\varvec{\theta }}_{{\rho _n}}) \ge \ell (\hat{\varvec{\theta }}_q). \end{aligned}$$

Combining (A.1), (A.4), (A.5), we obtain

$$\begin{aligned} P_{\mathrm{qmin}}(\hat{\varvec{\Lambda }}_{{\rho _n}})\le P_{\mathrm{qmin}}(\hat{\varvec{\Lambda }}_q)\rightarrow P_{\mathrm {qmin}}(\varvec{\Lambda }^{*}_q)\quad a.s. \end{aligned}$$

for some $(\mathrm{vec}(\varvec{\Lambda }^*_q)^T,\mathrm{diag}(\varvec{\Psi }^*_q)^T,\mathrm{vech}(\varvec{\Phi }^*_q)^T)^T\in \Theta ^*_q$. Therefore, we have

$$\begin{aligned} \mathop {\lim }_{n \rightarrow \infty } P_{\mathrm{qmin}}(\hat{\varvec{\Lambda }}_{\rho _n})\le P_{\mathrm {qmin}}(\varvec{\Lambda }^{*}_q)\quad a.s. \end{aligned}$$

As shown in (A.2), $\lim _{n\rightarrow \infty } d(\hat{\varvec{\theta }}_{\rho _n},\Theta _*)=0\;\;a.s.$, and $\varvec{\Lambda }^{*}_q$ is a minimizer of $P_{\mathrm{qmin}}(\cdot )$ over $\Theta _*$, so that the proof is complete.

## Detail of the Algorithm

### Update Equation of EM Algorithm for Fixed Tuning Parameters

We provide update equations of factor loadings and unique variances when $\rho$ and $\gamma$ are fixed. Suppose that $\varvec{\Lambda }_{\mathrm{old}}$, $\varvec{\Psi }_{\mathrm{old}}$, and $\varvec{\Phi }_{\mathrm{old}}$ are the current values of parameters. The parameter can be updated by minimizing the negative expectation of the complete-data penalized log-likelihood function with respect to $\varvec{\Lambda }$, $\varvec{\Psi }$, and $\varvec{\Phi }$ (e.g., Hirose and Yamamoto, 2014):

A.6 $$\begin{aligned} Q(\varvec{\Lambda },\varvec{\Psi }, \varvec{\Phi })= & {} \sum _{i = 1}^p \left( \log \psi _i + \frac{s_{ii} - 2\varvec{\lambda }_i^T\varvec{b}_i+ \varvec{\lambda }_i^T \varvec{A}\varvec{\lambda }_i}{\psi _i} \right) + \log |\varvec{\Phi }| + \mathrm{tr} ( \varvec{\Phi }^{-1} \varvec{A}) \nonumber \\&+ \rho P( \varvec{\Lambda })+C, \end{aligned}$$

where *C* is a constant and

A.7 $$\begin{aligned} \varvec{A}= & {} \varvec{M} ^{-1} + \varvec{M}^{-1}\varvec{\Lambda }_{\mathrm{old}}^T\varvec{\Psi }_{\mathrm{old }}^{-1}\varvec{S}\varvec{\Psi }_{\mathrm{old }}^{-1}\varvec{\Lambda }_{\mathrm{old }}\varvec{M}^{-1}, \end{aligned}$$

A.8 $$\begin{aligned} \varvec{b}_i= & {} \varvec{M}^{-1}\varvec{\Lambda }_{\mathrm{old}}^T\varvec{\Psi }_{\mathrm{old }}^{-1}\varvec{s}_i, \end{aligned}$$

A.9 $$\begin{aligned} \varvec{M}= & {} \varvec{\Lambda }_{\mathrm{old }}^T\varvec{\Psi }_{\mathrm{old }}^{-1}\varvec{\Lambda }_{\mathrm{old }} + \varvec{\Phi }_{\mathrm{old}}^{-1}. \end{aligned}$$

Here, $\varvec{s}_i$ is the *i*th column vector of $\varvec{S}$.

In practice, minimization of (A.6) is difficult, because the prenet penalty consists of nonconvex functions. Therefore, we use a coordinate descent algorithm to obtain updated loading matrix $\varvec{\Lambda }_{\mathrm{new}}$. Let $\tilde{{\lambda }}_{i}^{(j)}$ be a ($m-1$)-dimensional vector $( {\tilde{\lambda }}_{i1},{\tilde{\lambda }}_{i2},\dots ,{\tilde{\lambda }}_{i(j-1)},{\tilde{\lambda }}_{i(j+1)},\dots ,{\tilde{\lambda }}_{im})^T$. The parameter $\lambda _{ij}$ can be updated by maximizing (A.6) with the other parameters $\tilde{{\lambda }}_{i}^{(j)}$ and with $\varvec{\Psi }$ being fixed, that is, we solve the following problem.

A.10 $$\begin{aligned} {\tilde{\lambda }}_{ij}= & {} \mathrm{arg} \min _{\lambda _{ij}} \frac{1}{2\psi _i} \left\{ a_{jj}\lambda _{ij}^2 - 2\left( b_{ij} - \sum _{k \ne j} a_{kj} {\tilde{\lambda }}_{ik} \right) \lambda _{ij} \right\} \nonumber \\&+ \rho \left[ \left\{ \frac{1}{2} (1-\gamma ) \sum _{k \ne j} {\tilde{\lambda }}_{ik}^2\right\} \lambda _{ij}^2 + \left( \gamma \sum _{k \ne j} |{\tilde{\lambda }}_{ik}| \right) |\lambda _{ij}| \right] \nonumber \\= & {} \mathrm{arg} \min _{\lambda _{ij}} \frac{1}{2\psi _i} \left\{ (a_{jj}+\beta )\lambda _{ij}^2 - 2\left( b_{ij} - \sum _{k \ne j} a_{kj} {\tilde{\lambda }}_{ik} \right) \lambda _{ij} \right\} + \rho \xi |\lambda _{ij}| \nonumber \\= & {} \mathrm{arg} \min _{\lambda _{ij}}\frac{1}{2} \left( \lambda _{ij} - \frac{b_{ij} - \sum _{k \ne j} a_{kj} {\tilde{\lambda }}_{ik} }{a_{jj} + \beta } \right) ^2 + \frac{\psi _i\rho \xi }{a_{jj}+\beta } |\lambda _{ij}|, \end{aligned}$$

where

$$\begin{aligned} \beta= & {} \rho \psi _i(1-\gamma ) \sum _{k \ne j} {\tilde{\lambda }}_{ik}^2, \\ \xi= & {} \gamma \sum _{k \ne j} |{\tilde{\lambda }}_{ik}|. \end{aligned}$$

This is equivalent to minimizing the following penalized squared error loss function.

A.11 $$\begin{aligned} S({\tilde{\theta }}) = \mathrm{arg} \min _{\theta } \left\{ \frac{1}{2}(\theta - {\tilde{\theta }})^2 + \rho ^* |\theta | \right\} . \end{aligned}$$

The solution $S({\tilde{\theta }})$ can be expressed in a closed form using the soft thresholding function:

A.12 $$\begin{aligned} S({\tilde{\theta }})= \mathrm{sgn}({\tilde{\theta }}) (|{\tilde{\theta }} |- \rho ^*)_+, \end{aligned}$$

where $$A_+ = \max (A,0)$$.

For given $\varvec{\Lambda }_{\mathrm{new}}$, the parameters of unique variances and factor correlations, say $\varvec{\Psi }_{\mathrm{new}}$ and $\mathbf {\Phi }_{\mathrm{new}}$, are expressed as

$$\begin{aligned} \psi _i^{\mathrm{new}}= & {} s_{ii} - 2 (\varvec{\lambda }^{\mathrm{new}}_i)^T\varvec{b}_i + (\varvec{\lambda }^{\mathrm{new}}_i)^T \varvec{A} \varvec{\lambda }^{\mathrm{new}}_i \quad (i = 1,\dots ,p),\\ \mathbf {\Phi }_{\mathrm{new}}= & {} \mathop {\mathrm{arg~min}}\limits _{\mathbf {\Phi }} \{ \log |\mathbf {\Phi }| + \mathrm{tr}(\mathbf {\Phi }^{-1}{\mathbf {A}}) \}, \end{aligned}$$

where $\psi _i^{\mathrm{new}}$ is the *i*th diagonal element of $\varvec{\Psi }_{\mathrm{new}}$, and $\varvec{\lambda }^{\mathrm{new}}_i$ is the *i*th row of $\varvec{\Lambda }_{\mathrm{new}}$. The new value $\mathbf {\Phi }_{\mathrm{new}}$ may not be expressed in an explicit form, because all of the diagonal elements of $\mathbf {\Phi }$ are fixed by 1. Thus, $\mathbf {\Phi }_{\mathrm{new}}$ is obtained by the Broyden–Fletcher–Goldfarb–Shanno optimization procedure.

### Algorithm Complexity

The complexity for our proposed algorithm for the orthogonal case is considered. To update $\varvec{\Lambda }$, we need a matrix $\varvec{A}$ in (A.7). The matrix computation of $\varvec{A}$ requires $O(p^2m)$ operation (Zhao et al., 2007). In the coordinate descent algorithm, we need to compute ${\tilde{\theta }}$ in (A.11) for each step, in which *O*(*m*) operation is required. For simplicity, we consider the case where the number of cycles of the coordinate descent algorithm is one. We remark that our algorithm converges to the (local) optima for this case because both EM and coordinate descent algorithms monotonically decrease the objective function at each iteration. In this case, we need *O*(*m*) to update $\lambda _{ij}$ ($i=1,\dots ,p$; $j=1,\dots ,m$), and therefore, $O(pm^2)$ operation is required to update $\varvec{\Lambda }$ in the coordinate descent algorithm. As a result, the algorithm complexity to update $\varvec{\Lambda }$ is $O(p^2m) + O(pm^2) = O(p^2m)$.

For the update of $\varvec{\Psi }$, we need $O(pm^2)$ operation because the computation of $(\varvec{\lambda }^{\mathrm{new}}_i)^T \varvec{A} \varvec{\lambda }^{\mathrm{new}}_i$ in (A.13) requires $O(m^2)$ operation for $i=1,\dots ,p$. Note that when the unpenalized maximum likelihood estimation is conducted, the order *O*(*pm*) is achieved (Zhao et al., 2007); however, this order may not be achieved for the prenet penalization.
