# Supplementary material for: Sparse and Simple Structure Estimation via Prenet Penalization
Source: Psychometrika. 2022 May 23;88(4):1381–406. doi: 10.1007/s11336-022-09868-4 (PMC10656346; doi:10.1007/s11336-022-09868-4)
Supplement: Supplementary file 1 — (pdf 1749 KB) [file 11336_2022_9868_MOESM1_ESM.pdf]

Supplemental Material for sparse and simple structure estimation via prenet penalization

Kei Hirose and Yoshikazu Terada

S1 Further analysis for simulation data

S1.1 Comparison of prenet and elastic net for small model that is similar to Model (B) in Section 6.1

To illustrate the usefulness of the prenet, we provide loading matrices estimated by the prenet and the elastic net based on the data generated by a dense loading matrix. Suppose that the true loading matrix is

$$\mathbf{\Lambda}_d = \begin{pmatrix} 0.90 & 0.80 & 0.70 & 0.18 & 0.28 & 0.08 \\ 0.11 & 0.24 & 0.29 & 0.90 & 0.80 & 0.70 \end{pmatrix}^T. \tag{S1.1}$$

Here, "d" in $\mathbf{\Lambda}_d$ denotes dense because the loading matrix does not include zero values. In contrast, many simulation studies of the factor model (e.g., Lopes and West 2004) assume that some of the true factor loadings are exactly zero, as follows.

$$\mathbf{\Lambda}_s = \begin{pmatrix} 0.90 & 0.80 & 0.70 & 0.00 & 0.00 & 0.00 \\ 0.00 & 0.00 & 0.00 & 0.90 & 0.80 & 0.70 \end{pmatrix}^T. \tag{S1.2}$$

Here, "s" in $\mathbf{\Lambda}_s$ denotes sparse. In this numerical example, we use $\mathbf{\Lambda}_d$ instead of $\mathbf{\Lambda}_s$ as a true loading matrix. This is because, in many applications, some of the factor loadings can be nearly—but not exactly—zero.

With the penalization procedure, we expect that

(i) for large ρ, the estimated loading matrix is close to (S1.2),

(ii) for small ρ, we obtain an estimate that is close to (S1.1).

Table S1.1 shows the loading matrices estimated by the elastic net for various values of ρ. The factor correlation is fixed as $\mathbf{\Phi} = \boldsymbol{I}_m$, that is, orthogonal factor model is considered. With the lasso penalty (i.e., $\gamma = 1$), when $\rho > 0.29$, we obtain a one-factor model; the largest value that

Table S1.1: Loading matrices estimated by the *elastic net* for various values of ρ.

	$\gamma = 1$						$\gamma = 0.01$					
	$\rho = 0.29$		$\rho = 0.1$		$\rho = 0.01$		$\rho = 1.0$		$\rho = 0.1$		$\rho = 0.01$	
	F1	F2	F1	F2	F1	F2	F1	F2	F1	F2	F1	F2
V1	0.51	0.00	0.63	0.00	0.73	0.02	0.44	0.21	0.58	0.34	0.58	0.36
V2	0.78	0.00	0.88	0.00	0.98	0.00	0.46	0.30	0.77	0.45	0.80	0.47
V3	0.40	0.10	0.51	0.18	0.64	0.24	0.30	0.30	0.40	0.47	0.39	0.49
V4	0.22	0.50	0.34	0.58	0.49	0.64	0.03	0.49	0.07	0.73	0.08	0.75
V5	0.17	0.58	0.29	0.65	0.44	0.71	0.00	0.51	0.00	0.77	0.00	0.79
V6	-0.00	0.53	0.07	0.65	0.21	0.74	-0.05	0.39	-0.18	0.67	-0.19	0.69

Table S1.2: Loading matrices estimated by the *prenet* for various values of ρ.

	$\gamma = 1$						$\gamma = 0.01$					
	$\rho = 0.4$		$\rho = 0.2$		$\rho = 0.01$		$\rho = 43$		$\rho = 0.5$		$\rho = 0.02$	
	F1	F2	F1	F2	F1	F2	F1	F2	F1	F2	F1	F2
V1	0.84	0.00	0.72	0.00	0.74	0.02	0.84	0.00	0.71	0.11	0.72	0.17
V2	0.89	0.00	0.96	0.00	0.99	0.00	0.89	0.00	0.92	0.15	0.97	0.19
V3	0.74	0.00	0.57	0.19	0.65	0.24	0.74	0.00	0.55	0.30	0.59	0.37
V4	0.00	0.82	0.32	0.63	0.50	0.64	0.00	0.82	0.30	0.70	0.38	0.74
V5	0.00	0.89	0.25	0.71	0.45	0.72	0.00	0.89	0.23	0.77	0.31	0.80
V6	0.00	0.73	0.00	0.75	0.21	0.75	0.00	0.73	0.02	0.75	0.07	0.78

provides a two-factor model with the lasso is $\rho = 0.29$. In this case, $\hat{\lambda}_{32}$, $\hat{\lambda}_{41}$, and $\hat{\lambda}_{51}$ are nonzero, which means (i) is not satisfied. When ρ is small, $\hat{\lambda}_{12}$, $\hat{\lambda}_{22}$, and $\hat{\lambda}_{32}$ are smaller than true values, and $\hat{\lambda}_{41}$, $\hat{\lambda}_{51}$, and $\hat{\lambda}_{61}$ become much larger than the true values. Estimating some coefficients toward exactly or nearly zero makes other small coefficients larger than expected. As a result, (ii) is not satisfied with the lasso. When $\gamma = 0.01$, we obtain similar results, and thus, the ridge penalty does not make any contribution to the approximation of the true loading matrix.

The loading matrices estimated by the prenet penalty are provided in Table S1.2. The prenet with $\gamma = 1$ can produce a solution that is very close to (S1.2) for large ρ. When ρ is small, however, we obtain a tendency similar to the lasso; $\hat{\lambda}_{41}$, $\hat{\lambda}_{51}$, and $\hat{\lambda}_{61}$ are larger than the true values. Therefore, (i) is satisfied but (ii) is not when $\gamma = 1$. When $\gamma = 0.01$, we obtain a loading matrix that is similar to (S1.2) for large ρ. Furthermore, as ρ reduces, we obtain a loading matrix that is close to the true loading matrix in (S1.1). Thus, the prenet penalty with $\gamma = 0.01$ satisfies both (i) and (ii).

S1.2 Further investigation into the elastic net

The result of Section S1.1 shows that the elastic net yields poor performance. Meanwhile, one reviewer pointed out that there is a recent publication by Scharf & Nestler (2019) in which the elastic net penalty does perform quite well numerically. We further investigate the cause of the inconsistent results through further simulation.

Our simulation setting is compared with Scharf & Nestler's (2019) one. We observe that a significant difference lies in the assumption of the factor correlation. In Section S1.1, the estimation is done under the assumption that each factor is orthogonal. On the other hand, Scharf & Nestler (2019) assume the oblique model; these authors estimate the factor correlation along with other parameters via the penalized maximum likelihood procedure. Therefore, it is interesting to compare the estimation accuracy of the orthogonal model and that of the oblique model.

We generate data from (S1.1) with true value of factor correlation being 0 and compare the factor loadings estimated by orthogonal and oblique models. The estimation is done by elastic net with $\gamma = 0.01$. The regularization parameter ρ is selected by the BIC. The loading matrices for $n = 50, 100, 500$ are presented in Table S1.3. Because the true factor correlation is 0, we

Table S1.3: Factor loadings estimated by both orthogonal and oblique models for simulation model in (S1.1).

	Orthogonal model						Oblique model					
	$n = 50$		$n = 100$		$n = 500$		$n = 50$		$n = 100$		$n = 500$	
	F1	F2	F1	F2	F1	F2	F1	F2	F1	F2	F1	F2
V1	0.61	0.43	0.89	0.37	0.93	0.00	0.71	0.11	0.93	0.00	0.92	0.08
V2	0.84	0.54	0.61	0.40	0.83	0.16	0.97	0.11	0.65	0.13	0.79	0.23
V3	0.41	0.57	0.59	0.43	0.72	0.21	0.56	0.33	0.63	0.17	0.68	0.27
V4	0.08	0.83	0.05	0.95	0.28	0.85	0.31	0.71	0.15	0.87	0.13	0.87
V5	0.00	0.86	0.17	0.83	0.39	0.75	0.24	0.78	0.26	0.70	0.26	0.78
V6	-0.21	0.76	0.00	0.76	0.12	0.70	0.00	0.78	0.08	0.70	0.00	0.71

expect that the orthogonal model would perform better than oblique model. Nevertheless, with orthogonal model, the elastic net cannot approximate the true cross-loadings (small loadings), especially when n is small. Indeed, $\hat{\lambda}_{41}$, $\hat{\lambda}_{51}$, and $\hat{\lambda}_{61}$ are smaller than true values, while $\hat{\lambda}_{12}$, $\hat{\lambda}_{22}$, and $\hat{\lambda}_{32}$ are larger than true values when $n = 50$ and $n = 100$. This phenomenon is also found in Example 3.1 of Hirose and Yamamoto (2014); the lasso-type regularization tends to estimate bi-factor model when the true cross-loadings are nonzero.

Meanwhile, for the oblique model, the magnitudes of cross-loadings are appropriately captured. The estimation accuracy is improved as the number of observations becomes large. All cross-loadings are slightly smaller than true values because of the assumption of factor correlation; indeed, the estimate of factor correlation is 0.18 when $n = 50$. We remark that all primary loadings are positive and cross-loadings are positive as well, and therefore, the overlap is represented as the factor correlation parameter.

We also compare the performance of the orthogonal model and that of oblique model with a loading matrix given by Schmitt and Sass (2011); Scharf and Nestler (2019). The "complex structure" in Schmitt and Sass (2011) is used, in which the cross-loadings are relatively large. We set $n = 100$ and generate data with the true factor correlation being 0. The loading matrix

Table S1.4: The loading matrices of true values, estimate for the orthogonal model, and estimate for the oblique model for simulation model in Schmitt and Sass (2011).

			Elastic net						Prenet					
True loadings			Orthogonal			Oblique			Orthogonal			Oblique		
F1	F2	F3	F1	F2	F3	F1	F2	F3	F1	F2	F3	F1	F2	F3
0.76	0.25	0.15	0.87	0.00	0.00	0.79	0.11	0.28	0.81	0.24	0.20	0.80	0.06	0.11
0.76	0.10	0.25	0.80	-0.05	-0.02	0.75	0.05	0.24	0.77	0.17	0.16	0.77	0.00	0.09
0.76	0.30	0.05	0.84	0.16	-0.20	0.81	0.26	0.06	0.78	0.38	-0.02	0.79	0.26	-0.13
0.77	0.46	0.10	0.81	0.23	-0.07	0.71	0.33	0.18	0.70	0.44	0.11	0.67	0.31	0.01
0.77	0.15	0.46	0.91	-0.03	0.26	0.73	0.07	0.54	0.79	0.21	0.46	0.73	-0.01	0.40
0.76	0.20	0.20	0.78	0.01	0.02	0.70	0.10	0.27	0.72	0.22	0.20	0.70	0.06	0.12
0.05	0.76	0.30	0.38	0.71	0.10	0.18	0.75	0.17	0.14	0.78	0.15	0.01	0.77	0.08
0.32	0.80	0.48	0.63	0.71	0.25	0.34	0.78	0.40	0.33	0.85	0.35	0.17	0.77	0.28
0.47	0.79	0.26	0.66	0.70	0.00	0.47	0.78	0.17	0.42	0.85	0.12	0.31	0.80	0.02
0.10	0.76	0.20	0.41	0.71	0.00	0.24	0.75	0.09	0.19	0.79	0.06	0.08	0.79	-0.01
0.05	0.75	0.05	0.18	0.77	-0.01	0.03	0.79	0.00	-0.04	0.78	-0.01	-0.15	0.84	-0.06
0.31	0.77	0.15	0.58	0.63	0.02	0.40	0.70	0.17	0.36	0.76	0.12	0.25	0.71	0.04
0.05	0.45	0.76	0.32	0.55	0.62	-0.05	0.57	0.65	0.00	0.60	0.64	-0.20	0.52	0.65
0.48	0.32	0.80	0.76	0.25	0.56	0.43	0.32	0.76	0.51	0.43	0.70	0.35	0.23	0.67
0.31	0.21	0.77	0.59	0.10	0.60	0.29	0.15	0.75	0.38	0.24	0.71	0.24	0.05	0.71
0.15	0.10	0.76	0.33	-0.02	0.71	0.02	0.00	0.78	0.14	0.06	0.77	0.00	-0.11	0.81
0.26	0.26	0.77	0.52	0.22	0.70	0.16	0.25	0.82	0.26	0.33	0.79	0.09	0.15	0.80
0.20	0.10	0.76	0.48	0.04	0.58	0.20	0.08	0.70	0.30	0.15	0.67	0.17	-0.02	0.68

is estimated by the elastic net with $\gamma = 0.01$. For comparison, we also apply the prenet with $\gamma = 0.01$. The loading matrices of true values, estimate for the orthogonal model, and estimate for the oblique model are shown in Table S1.4. The true loading matrix shows that each factor affects 6 variables. Some of the true values of cross-loadings are relatively large. With the elastic net, the orthogonal model results in almost bi-factor model; the estimate of the loading matrix

that is completely different from the true one. On the other hand, the oblique model is able to correctly detect the primary loadings. Therefore, the assumption of factor correlation plays an important role in estimating the simple structure even when the true factor correlations are 0. We note that the prenet performs well for both orthogonal and oblique models.

In summary, with the orthogonal model, the elastic net (and also the lasso) often estimates a loading matrix whose cross-loadings are larger than true values when there exist nonzero cross-loadings. To address this issue, one can assume the oblique model. However, incorporating the factor correlation leads to the estimates that are smaller than the true cross-loadings. We remark that the prenet performs well even when the orthogonal model is assumed.

S1.3 Stability of our proposed algorithm

The loss function of the prenet, given in (2), is nonconvex; the loss function has multiple local minima. Therefore, we use t random starts and select a model that minimizes the loss function. The relationship between the number of random starts and the stability of our proposed algorithm is investigated. We generate one dataset from Models (A) – (C) in Section 6.1, and estimate the solution path with t random initial values. Figures S1.1, S1.2, S1.3 depict the loss functions as a function of ρ over 100 runs for $n = 50$, 100, and 500, respectively. For each run, we set t initial values, optimize parameter, and select a parameter that minimizes the loss function. Thus, we employ the cold start. The result shows that the $t = 1$ initial value sometimes results in an unstable estimator. When $t = 10$, many stabler solutions are obtained but still, a few solutions are unstable. With $t = 100$, all solutions are stable. Therefore, it would be sufficient to prepare 100 random starts to get a stable solution in practical situations.

S1.4 Further results of the Monte Carlo simulation in Section 6.2

The true positive rate and false positive rate of the estimated loading matrix are depicted in Figures S1.4 and S1.5.

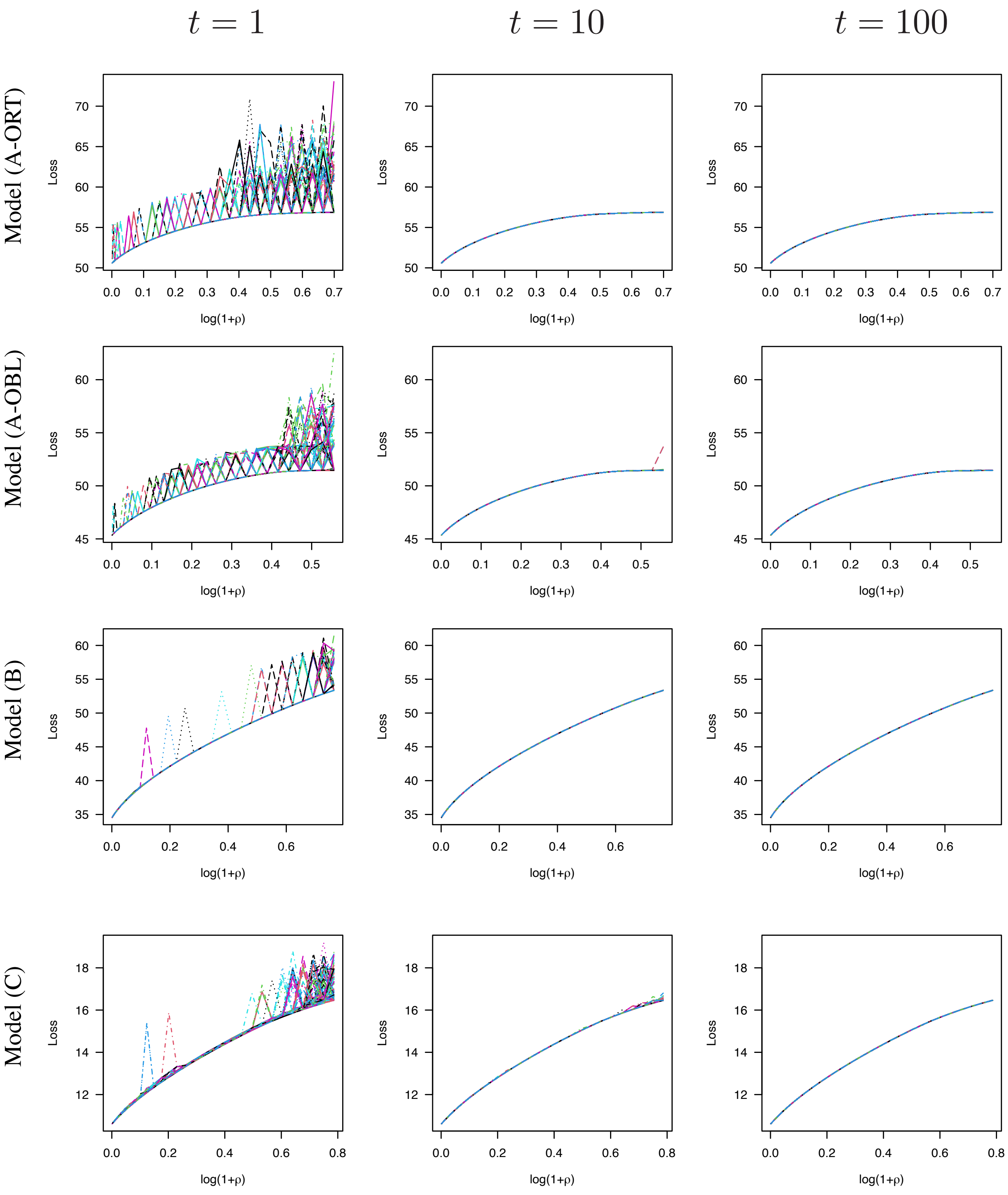

Figure S1.1: Loss functions as a function of $\log(1+\rho)$ over 100 runs when $n = 50$.

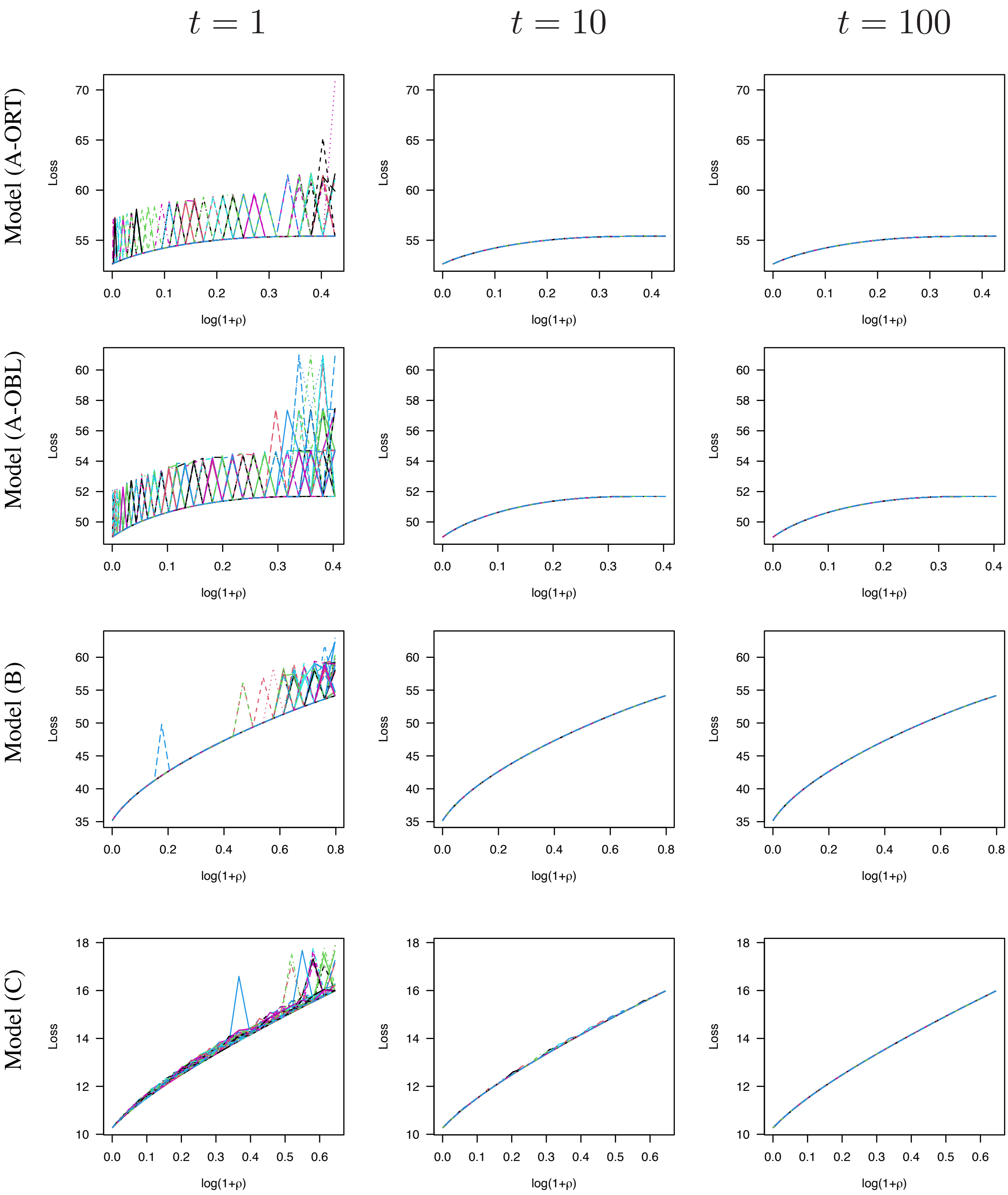

Figure S1.2: Loss functions as a function of $\log(1 + \rho)$ over 100 runs when $n = 100$.

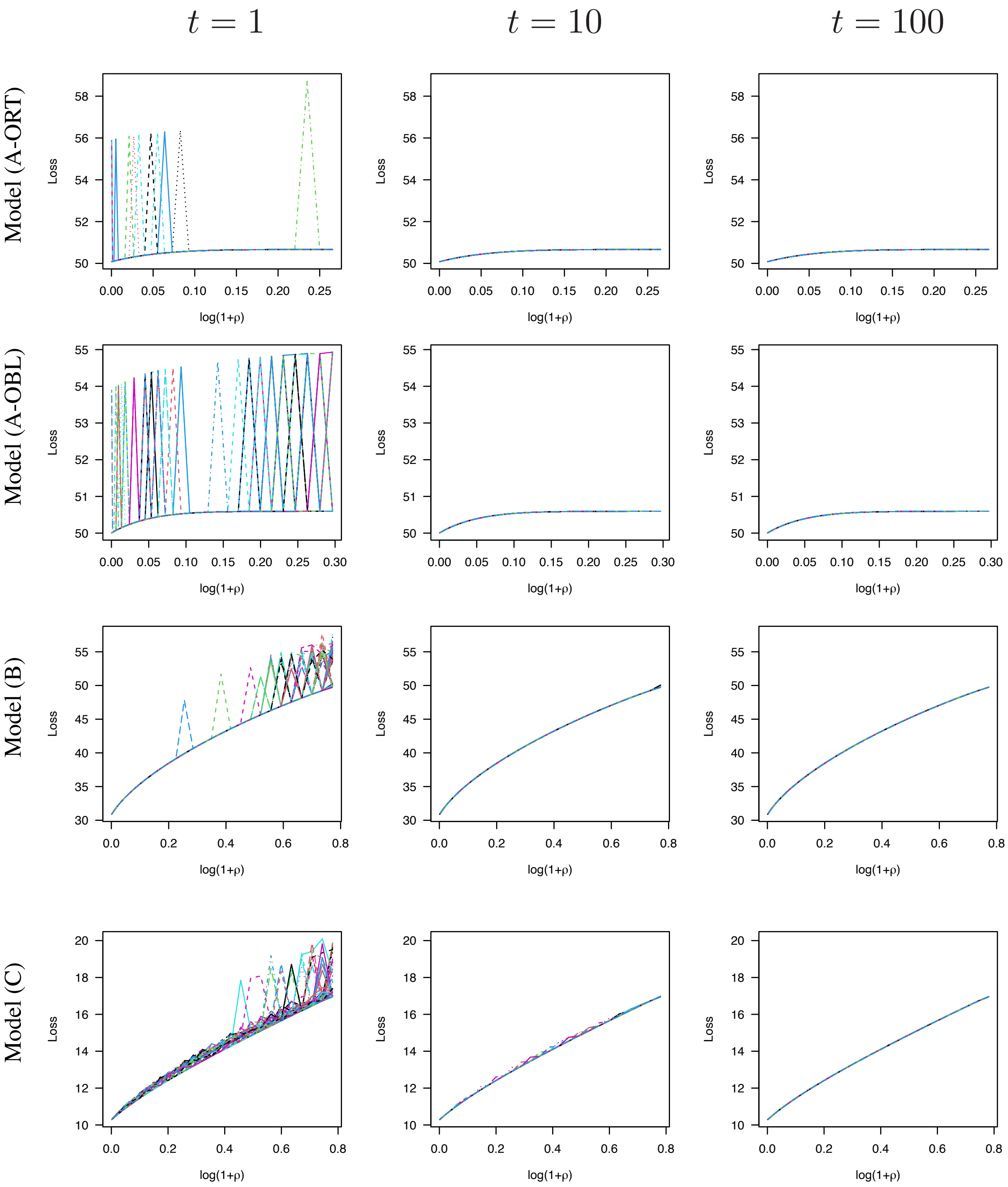

Figure S1.3: Loss functions as a function of $\log(1+\rho)$ over 100 runs when $n = 500$.

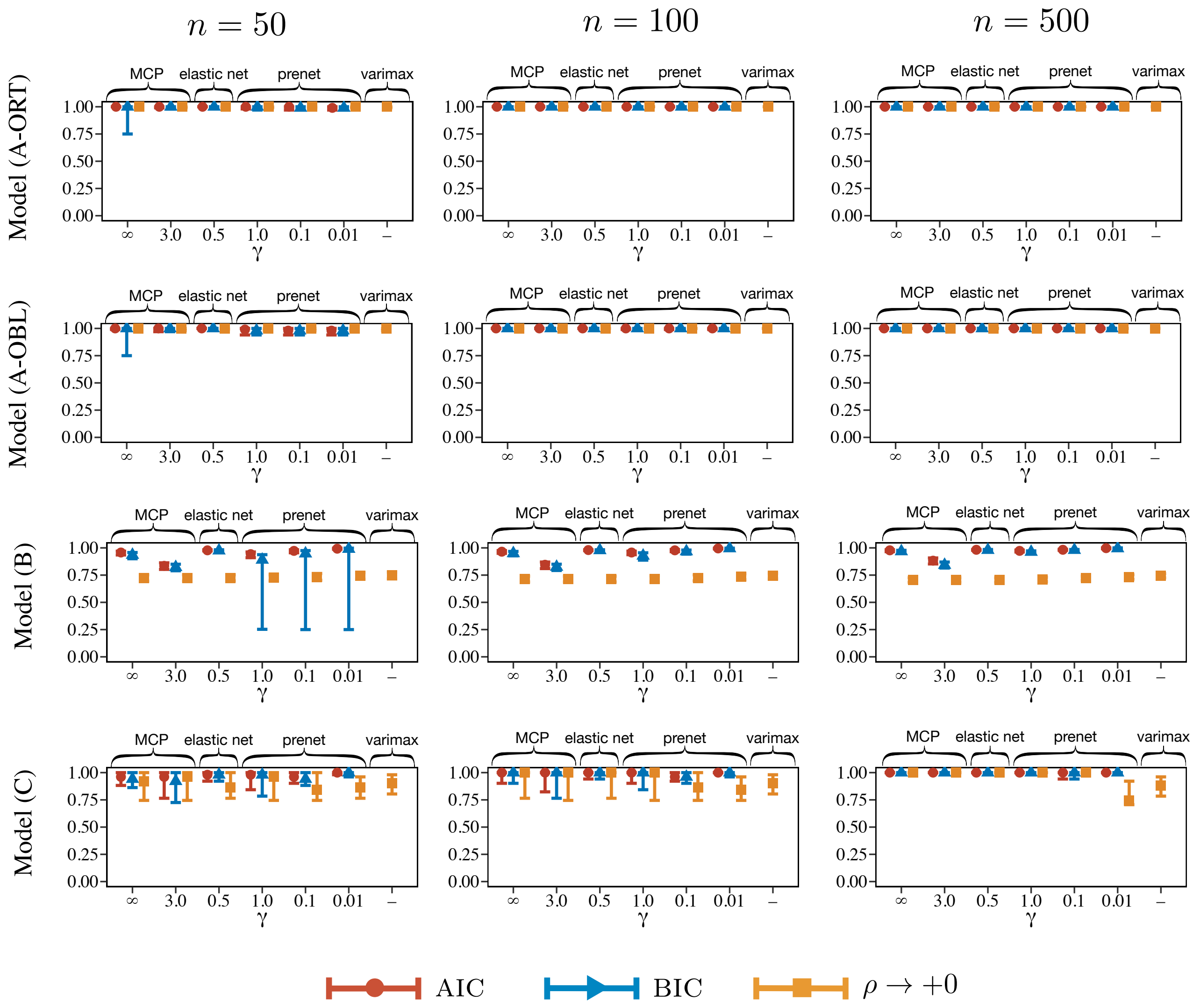

Figure S1.4: The true positive rate of the estimated loading matrix. The range of the error bar indicates 90 % confidence interval. Here, "$\rho \to +0$" denotes a limit of the estimate of the factor loadings, $\lim_{\rho \to +0} \hat{\Lambda}_\rho$, which corresponds to the factor rotation.

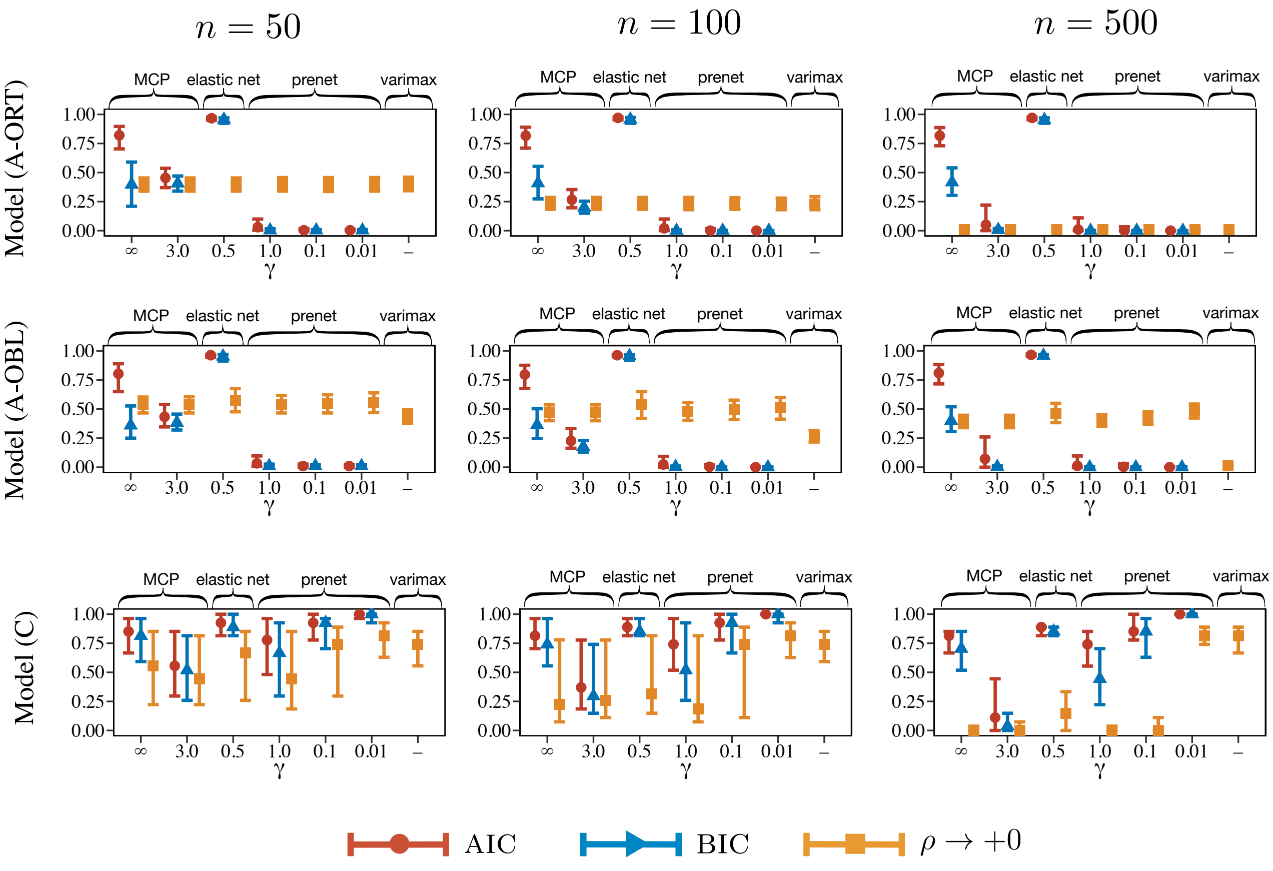

Figure S1.5: The false positive rate of the estimated loading matrix. The range of the error bar indicates 90 % confidence interval. The result for Model (B) is not presented because all of the true factor loadings are nonzero. Here, "$\rho \to +0$" denotes a limit of the estimate of the factor loadings, $\lim_{\rho \to +0} \hat{\Lambda}_\rho$, which corresponds to the factor rotation.

S2 Further analysis of real data

S2.1 Application to resting state fMRI data

We investigate a cluster structure of brain regions of interest using a resting state fMRI (rfMRI) data. We use a single-subject preprocessed rfMRI data in Human Connectome Project (`https://www.humanconnectome.org/`). The rfMRI data are acquired in a single run of 1200 time points (approximately 15 minutes). We view 268 brain regions proposed by Shen et al. (2013) as regions of interest, and aggregate the preprocessed voxel-wise rfMRI data into the 268 dimensional time series data for regions of interest by taking an average in the original region.

S2.1.1 Clustering

We conduct cluster analysis of the 268 regions of interest. Because the cluster analysis is unsupervised learning, it is difficult to define a true cluster. We consider target clusters as 8 clusters defined by Finn et al. (2015); the number of factors is set as $m = 8$. These 8 clusters are interpretable and determined by the group analysis of 126 subjects (Finn et al. 2015). On the other hand, we use a single-subject rfMRI data with 268 regions. We conduct a clustering by

- Ward's method based on correlations among 268 regions of interest,
- perfect simple structure estimation via prenet penalization with $\gamma = 1$.

We use $\xi_{ij} = 1 - |r_{ij}|$ as a dissimilarity between ith region and jth region on Ward's method, where r_{ij} is a correlation between time series of ith region and that of jth region.

Figure S2.6 shows the clusters defined by Finn et al. (2015) and the results of both Ward's method and prenet penalization. In each subfigure, the colored points are located at the center coordinates of the corresponding regions of interest. The same color is corresponding to same cluster so that colors of regions of interest represent clusters. On the results of Ward's method and prenet penalization, the colors combinations are chosen by matching the colors of clusters of Finn et al. (2015) as much as possible. In order to compare these results more precisely, we use the adjusted rand index (ARI), which is a measure of the similarity between two clustering results. The larger the value of adjusted rand index, the higher the similarity between two clustering results is. The values of adjusted rand index between the two clustering results are given as follows.

- Ward's method and definition of Finn et al. (2015): ARI = 0.177
- prenet penalization and definition of Finn et al. (2015): ARI = 0.208

Because the clusters defined by Finn et al. (2015) are interpretable, the result shows that the prenet penalization might result in more interpretable clusters than the Ward's method.

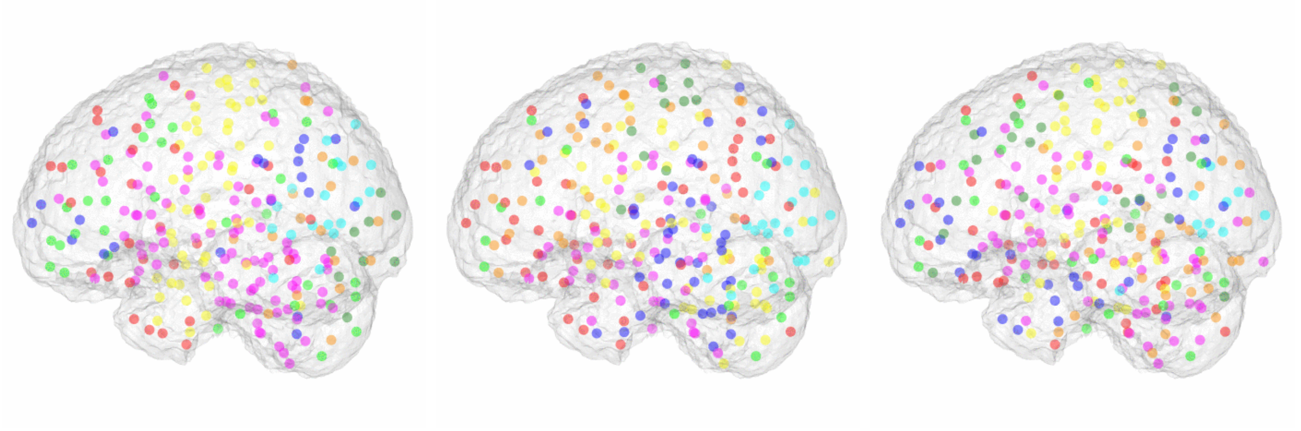

(a) Finn et al. (2015) (b) Ward's method (c) prenet with 8 factors

Figure S2.6: 8 clusters of 268 regions of interest.

S2.1.2 Reconstruction error

The performance of prenet is investigated by reconstruction error. We first divide the dataset into training and test datasets; the first and last 600 observations are regarded as training and test data, respectively. We compress test data with the parameter estimated by training data and compute the reconstruction error. For reconstruction of the penalized likelihood methods, including lasso, MCP with $\gamma = 3$, and prenet with $\gamma = 1$, the data are reconstructed via the posterior mean

$$\boldsymbol{\Lambda} E[\boldsymbol{F}_t|\boldsymbol{x}_t] = \boldsymbol{\Lambda}\boldsymbol{M}^{-1}\boldsymbol{\Lambda}^T\boldsymbol{\Psi}^{-1}\boldsymbol{x}_t \quad (t = 701, \dots, 1200). \tag{S2.3}$$

We also perform the k-means clustering and Ward's method. The data reconstruction of $\boldsymbol{x}_t$ is achieved using

$$\boldsymbol{\Lambda}(\boldsymbol{\Lambda}^T\boldsymbol{\Lambda})^{-1}\boldsymbol{\Lambda}^T\boldsymbol{x}_t \quad (t = 701, \dots, 1200), \tag{S2.4}$$

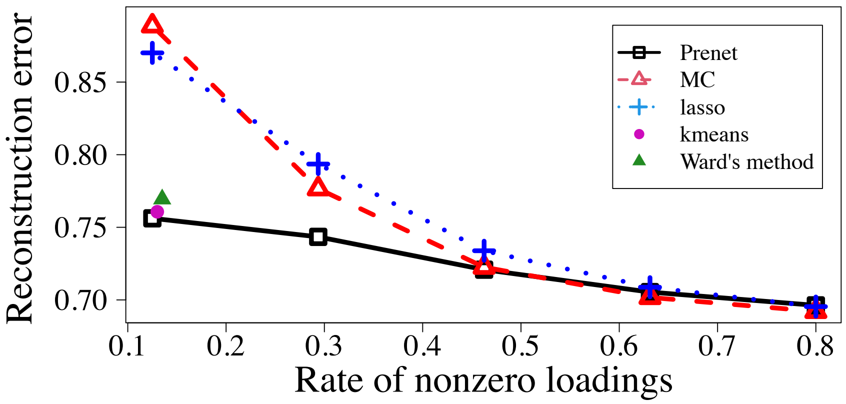

Figure S2.7: Reconstruction error as a function of the rate of nonzero loadings. The number of factors is $m = 8$. The horizontal positions for "k-means" and "Ward's method" are adjusted to find the overlapping objects easier.

where $\mathbf{\Lambda}$ is the estimated loading matrix given by (7).

The reconstruction error as a function of the rate of nonzero loadings is presented in Figure S2.7. The number of factors is $m = 8$. The prenet penalization performs the best when the rate of nonzero loadings is 0.125, in which the perfect simple structure estimation is achieved, thanks to Proposition 1. The k-means clustering and Ward's method also achieve good performance, but these methods cannot change the degrees of sparsity. The existing sparse estimations, the lasso and MCP, perform poorly for a small rate of nonzero loadings. We observe that the lasso and MCP result in a two-factor model; the last six column vectors of the loading matrix are estimated as zero vectors. As the rate of nonzero loadings increases, all of the penalization methods improve accuracy. For the high rate of nonzero loadings, the performance of existing penalization methods is comparable with that of the prenet.

S2.2 Application to electricity demand data

We apply our prenet penalty to electricity demand data collected from Tokyo Electric Power Company Holdings in Japan, available at `https://www.tepco.co.jp/en/forecast/html/download-e.html`. The dataset consists of electricity demand from January 1st, 2018, to December 31st, 2021. The demand is shown in MW at 1-hour intervals.

From a suppliers' point of view, it is crucial to interpret how electricity is used in daily life. For example, understanding the usage of electricity consumption would help develop strategies for energy-saving interventions (e.g., Guo et al. 2018) and demand response (e.g., Wang et al. 2018; Ruiz-Abellón et al. 2020). The basic idea of the demand response is that the customer tries to adjust the demand to the power from a supply. With the spread of renewable energy, the demand response has been becoming one of the essential research topics in the area of not only energy but also psychology (Good 2019; Lin et al. 2022). In addition, the COVID-19 changed the lifestyle from 2020 in Japan; it would be crucial to investigate the change in electricity usage patterns as a result of COVID-19.

First, we take a logarithm to the raw data and compute the first difference of the time series to eliminate strong positive autocorrelations. We then create 24-dimensional daily data for each year. Heatmaps of the correlation matrix on each year are depicted in Figure S2.8. The result shows that all correlation matrices are quite complex and also similar. Finding the characteristics of usage patterns of electricity demand from these correlation matrices would be a challenging problem.

We apply the prenet penalization, lasso, and MCP to each year's dataset. The number of factors is selected by the scree plot of factor analysis with cut-off eigenvalue being 1 (Guttman 1954; Kaiser 1960). The scree plot is implemented by `scree` function in `psych` package in `R`. For all years, a three-factor model is selected. The tuning parameter for the prenet and MCP are $\gamma = 1, 0.01$ and $\gamma = 3$, respectively. We employ the cold start with 100 starting values, which results in a reasonable estimate in terms of interpretation of the loading matrix. The regularization parameter ρ is selected by the BIC.

The heatmaps of the factor loadings estimated with the prenet are depicted in Figure S2.9. The results show that the estimates of the loading matrix are essentially identical to the value of γ. A rough interpretation of three factors might be given as follows: midnight, mealtimes, and working hours. The main loadings are similar each year. In particular, the results in 2018 and 2019 are almost identical. However, some of the cross-loadings are gradually changing from 2019 to 2021; for example, $\lambda_{6,3}$, $\lambda_{11,2}$, $\lambda_{12,2}$, and $\lambda_{19,3}, \lambda_{20,3}$. Here, the (i, j)th element of $\mathbf{\Lambda}$ is denoted as $\lambda_{i,j}$ instead of λ_{ij} throughout this section to present subscript of the factor loadings clearly.

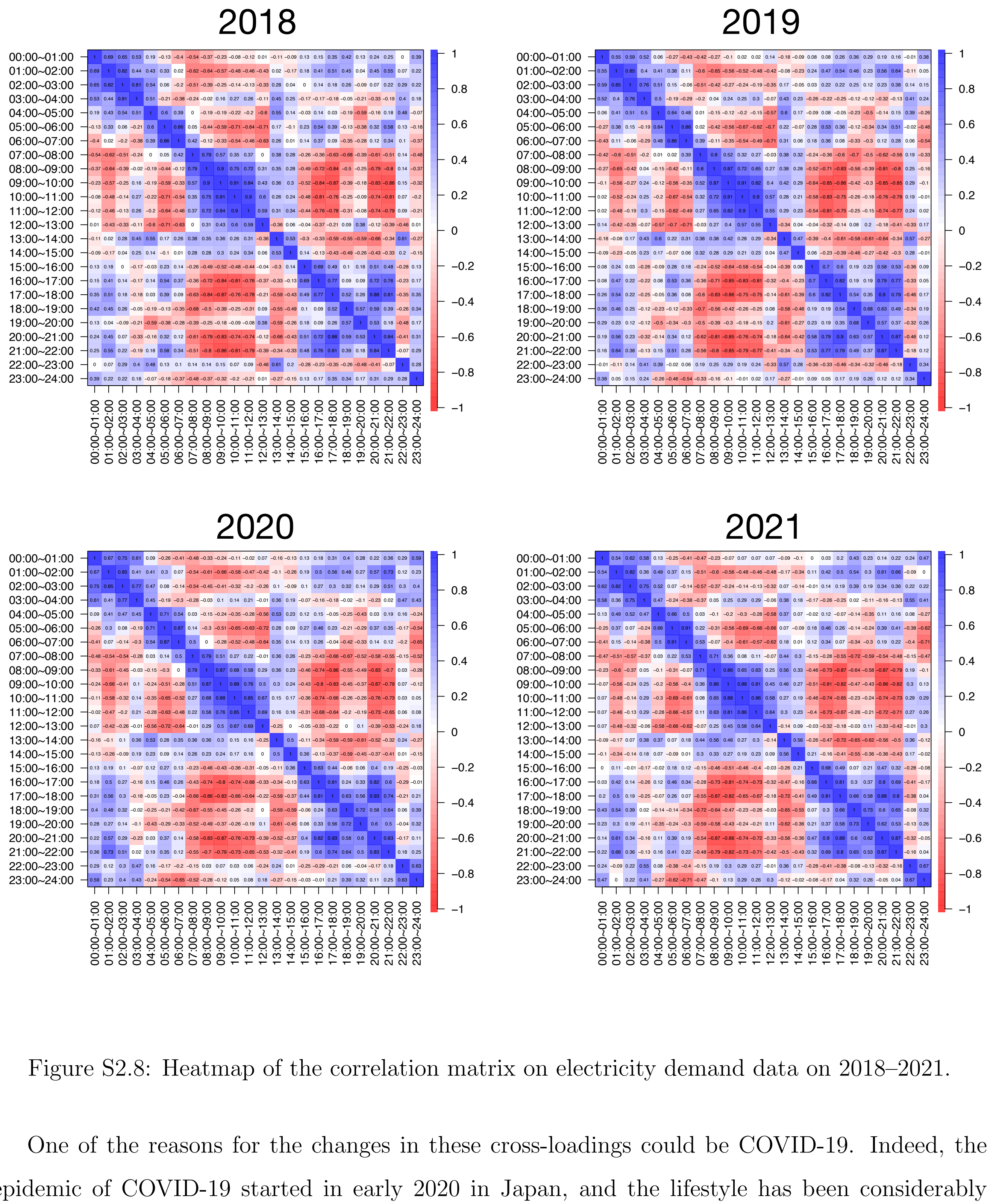

Figure S2.8: Heatmap of the correlation matrix on electricity demand data on 2018–2021.

One of the reasons for the changes in these cross-loadings could be COVID-19. Indeed, the epidemic of COVID-19 started in early 2020 in Japan, and the lifestyle has been considerably changed since then. The interpretation of latent factors might be useful to understand these

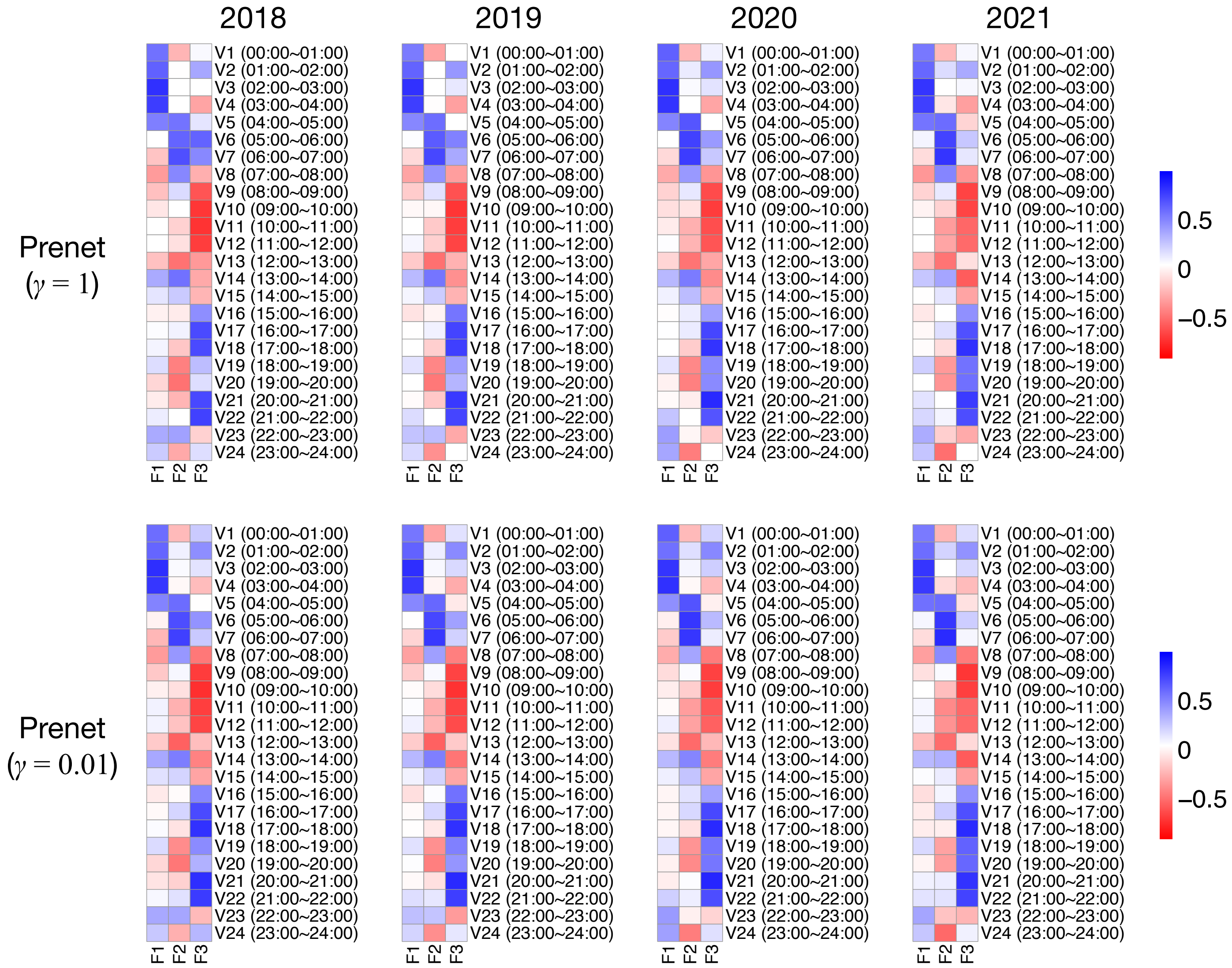

Figure S2.9: Heatmaps of the loading matrix estimated by prenet with $\gamma = 1$ and $\gamma = 0.01$ on electricity demand data on 2018–2021.

changes. For example, the electricity usage on the mealtimes factors corresponding to morning before the lunchtime ($\lambda_{11,2}$ and $\lambda_{12,2}$) and dinner time ($\lambda_{19,3}$ and $\lambda_{20,3}$) has changed because many people eat and work in the home due to the increase in telework (Long et al. 2021). A further investigation of these changes in factor loadings may be done by exploring individual households' electricity demand, but the dataset used in this analysis is the aggregate demand collected from Tokyo Electric Power Company Holdings. We would like to make a further investigation in the

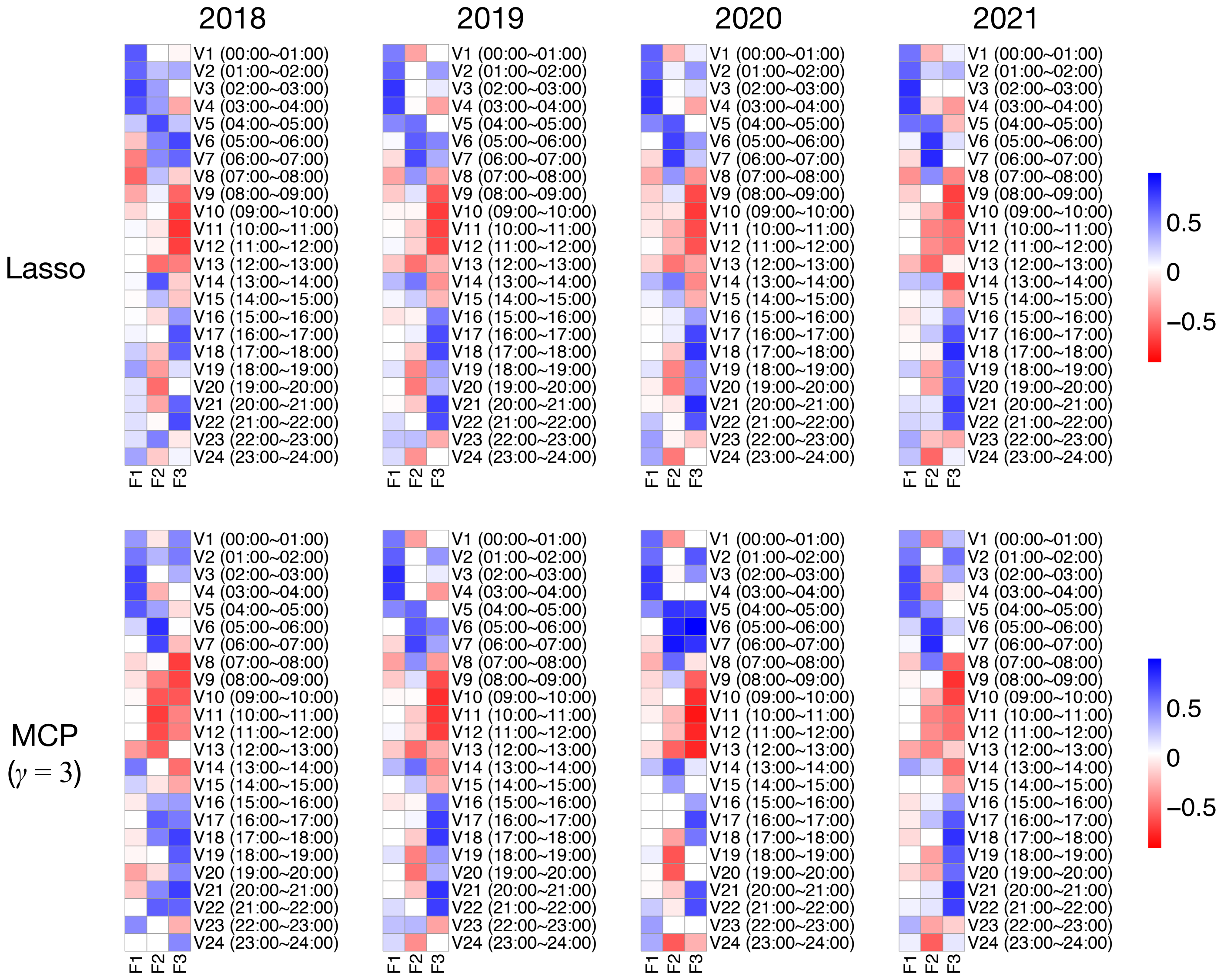

Figure S2.10: Heatmaps of the loading matrix estimated by lasso and MCP with $\gamma = 3$ on electricity demand data on 2018–2021.

future.

Figure S2.10 shows the heatmap of the loading matrix estimated with the lasso and MCP with $\gamma = 3$. The result shows that the estimate of the MCP changes much depending on the year, suggesting that MCP may result in unstable estimates. The lasso is stabler than the MCP but some of the factor loadings take different values between 2018 and 2019; see, e.g., $\lambda_{3,2}$, $\lambda_{4,2}$, $\lambda_{5,2}$,

$\lambda_{6,2}$, $\lambda_{7,2}$, and $\lambda_{7,3}$. With the prenet, these estimates do not change much from 2018 to 2019; therefore, the prenet may provide a stabler estimate than the MCP and the lasso.

S2.3 Loading matrix for big five personality traits data

The loading matrix for the big five personality traits data used in Section 7 is shown in Tables S2.5 and S2.6. The estimate is obtained by the prenet with $\gamma = 0.01$. The regularization parameter, ρ, is selected by the BIC.

S3 Comparison with Geomin criterion

This section compares the prenet penalty with Geomin criterion (Yates 1987). The Geomin criterion is expressed as

$$P_{\text{Geomin}}(\mathbf{\Lambda}) = \sum_{i=1}^{p} \left\{ \prod_{j=1}^{m} (\lambda_{ij}^2 + \varepsilon) \right\}^{\frac{1}{m}} = \sum_{i=1}^{p} \xi_i \tag{S3.5}$$

with some small nonnegative value ε. Here, $\xi_i := \left\{ \prod_{j=1}^{m} (\lambda_{ij}^2 + \varepsilon) \right\}^{\frac{1}{m}}$. Suppose that $\varepsilon = 0$. In this case, $\xi_i = 0$ if and only if *at least one element* out of $\{\lambda_{i1}, \dots, \lambda_{im}\}$ are exactly zero; therefore, the identifiability problem occurs. For example, when the loading matrix is given by Model (C) in the Monte Carlo simulation (Section 6), we have $P_{\text{Geomin}}(\mathbf{\Lambda}) = P_{\text{Geomin}}(\mathbf{\Lambda}\boldsymbol{T})$ with $\boldsymbol{T} = \begin{pmatrix} 1 & \mathbf{0}_3^T \\ \mathbf{0}_3 & \boldsymbol{T}_0 \end{pmatrix}$, where $\boldsymbol{T}_0$ is an arbitrary orthogonal matrix. Positive value of ε will handle this problem.

On the other hand, the prenet penalty is

$$P_{\text{prenet}}(\mathbf{\Lambda}) = \sum_{i=1}^{p} \tau_i, \quad \tau_i := \sum_{j=1}^{m-1} \sum_{k>j} \left\{ \gamma |\lambda_{ij}\lambda_{ik}| + \frac{1}{2}(1-\gamma)(\lambda_{ij}\lambda_{ik})^2 \right\}. \tag{S3.6}$$

With the prenet, $\tau_i = 0$ if and only if *all but one element* out of $\{\lambda_{i1}, \dots, \lambda_{im}\}$ are exactly zero. In this case, the identifiability problem that arises in the Geomin rotation does not occur. For example, it is shown that the prenet criterion is able to recover the perfect simple structure.

Interestingly, when $m = 2$, the Geomin criterion (S3.5) with $\varepsilon = 0$ is equivalent to the prenet criterion (S3.6) with $\gamma = 1$. In this case, the Geomin does not suffer from the identifiability problem.

Table S2.5: Loading matrix of the first 25 variables of big five data obtained by prenet penalization with $\gamma = 0.01$. The regularization parameter is selected by the BIC. The factor loadings whose absolute values are larger than 0.3 are written in bold.

	F1	F2	F3	F4	F5
E1: Am the life of the party.	**0.707**	-0.017	-0.002	-0.011	0.019
E2: Feel little concern for others.	**-0.720**	-0.037	0.074	0.007	-0.000
E3: Am always prepared.	**0.675**	-0.233	-0.192	0.106	-0.029
E4: Get stressed out easily.	**-0.762**	0.092	0.017	-0.016	0.007
E5: Have a rich vocabulary.	**0.755**	-0.051	-0.162	0.078	0.046
E6: Don't talk a lot.	**-0.604**	0.041	0.123	-0.026	-0.170
E7: Am interested in people.	**0.747**	-0.078	-0.110	0.025	-0.001
E8: Leave my belongings around.	**-0.575**	0.007	-0.054	0.100	-0.057
E9: Am relaxed most of the time.	**0.652**	-0.025	0.040	-0.059	0.126
E10: Have difficulty understanding abstract ideas.	**-0.687**	0.143	0.036	-0.021	-0.012
N1: Feel comfortable around people.	-0.110	**0.691**	-0.113	0.013	-0.076
N2: Insult people.	0.099	**-0.534**	0.009	-0.058	0.065
N3: Pay attention to details.	-0.130	**0.612**	-0.186	0.057	-0.029
N4: Worry about things.	0.190	**-0.415**	0.042	0.109	-0.035
N5: Have a vivid imagination.	-0.060	**0.531**	0.001	-0.042	-0.126
N6: Keep in the background.	-0.063	**0.758**	-0.041	-0.055	-0.077
N7: Sympathize with others' feelings.	-0.018	**0.724**	0.052	-0.128	0.020
N8: Make a mess of things.	-0.032	**0.754**	0.050	-0.120	0.012
N9: Seldom feel blue.	-0.063	**0.711**	0.164	-0.010	-0.031
N10: Am not interested in abstract ideas.	-0.290	**0.611**	-0.003	-0.145	0.066
A1: Start conversations.	-0.044	0.035	**0.523**	-0.013	-0.028
A2: Am not interested in other people's problems.	**0.341**	-0.062	**-0.525**	-0.020	0.070
A3: Get chores done right away.	0.092	0.252	**0.405**	-0.182	0.083
A4: Am easily disturbed.	0.046	0.028	**-0.798**	0.028	-0.000
A5: Have excellent ideas.	-0.138	-0.000	**0.656**	0.014	-0.000

Table S2.6: Loading matrix of the last 25 variables of big five data obtained by prenet penalization with $\gamma = 0.01$. The regularization parameter is selected by the BIC. The factor loadings whose absolute values are larger than 0.3 are written in bold.

	F1	F2	F3	F4	F5
A6: Have little to say.	0.020	0.101	**-0.632**	0.022	-0.088
A7: Have a soft heart.	**-0.317**	0.089	**0.615**	0.008	-0.010
A8: Often forget to put things back in their proper place.	0.137	-0.049	**-0.592**	0.102	0.019
A9: Get upset easily.	0.106	0.085	**-0.713**	0.051	0.051
A10: Do not have a good imagination.	**0.347**	-0.164	**-0.375**	0.116	0.082
C1: Talk to a lot of different people at parties.	0.019	-0.115	-0.008	**0.608**	0.097
C2: Am not really interested in others.	0.025	0.111	-0.044	**-0.571**	0.117
C3: Like order.	-0.027	-0.019	-0.074	**0.408**	0.253
C4: Change my mood a lot.	-0.083	**0.365**	0.033	**-0.548**	0.022
C5: Am quick to understand things.	0.092	-0.108	-0.060	**0.639**	-0.064
C6: Don't like to draw attention to myself.	-0.017	0.175	-0.000	**-0.611**	0.064
C7: Take time out for others.	-0.038	0.051	-0.038	**0.605**	0.029
C8: Shirk my duties.	-0.074	0.221	0.132	**-0.477**	-0.057
C9: Have frequent mood swings.	0.064	0.028	-0.086	**0.607**	-0.036
C10: Use difficult words.	0.018	-0.031	-0.048	**0.469**	0.249
O1: Don't mind being the center of attention.	0.000	-0.027	0.017	0.026	**0.613**
O2: Feel others' emotions.	-0.020	0.195	0.033	0.026	**-0.559**
O3: Follow a schedule.	0.043	0.092	-0.083	-0.115	**0.530**
O4: Get irritated easily.	0.000	0.099	0.128	0.089	**-0.481**
O5: Spend time reflecting on things.	0.212	-0.069	0.023	0.125	**0.568**
O6: Am quiet around strangers.	-0.097	0.036	0.099	0.067	**-0.482**
O7: Make people feel at ease.	0.062	-0.142	0.005	0.163	**0.485**
O8: Am exacting in my work.	-0.015	0.068	0.063	-0.040	**0.588**
O9: Often feel blue.	-0.140	0.142	-0.202	0.028	**0.343**
O10: Am full of ideas.	0.191	-0.012	-0.023	-0.012	**0.648**

If we make a Geomin-like modification to the prenet, the penalty may be expressed as

$$P(\mathbf{\Lambda}) = \sum_{i=1}^{p}\sum_{j=1}^{m-1}\sum_{k>j}\left\{\gamma(|\lambda_{ij}|+\varepsilon)(|\lambda_{ik}|+\varepsilon)| + \frac{1}{2}(1-\gamma)(\lambda_{ij}^2+\varepsilon)(\lambda_{ik}^2+\varepsilon)\right\}.$$

With a simple calculation, the above penalty turns out to be the weighted sum of the prenet and elastic net, which implies Proposition 1 does not hold. For this reason, it is better not to add a small positive value to the prenet penalty.

References

E. S. Finn, X. Shen, D. Scheinost, M. D. Rosenberg, J. Huang, M. M. Chun, X. Papademetris, and R. T. Constable. Functional connectome fingerprinting: identifying individuals using patterns of brain connectivity. *Nature neuroscience*, 18(11):1664–1671, 2015.

N. Good. Using behavioural economic theory in modelling of demand response. *Applied energy*, 239:107–116, 2019.

Z. Guo, K. Zhou, C. Zhang, X. Lu, W. Chen, and S. Yang. Residential electricity consumption behavior: Influencing factors, related theories and intervention strategies. *Renewable and Sustainable Energy Reviews*, 81:399–412, 2018.

L. Guttman. Some necessary conditions for common-factor analysis. *Psychometrika*, 19(2):149–161, 1954.

K. Hirose and M. Yamamoto. Estimation of an oblique structure via penalized likelihood factor analysis. *Computational Statistics & Data Analysis*, 79:120–132, 2014.

H. F. Kaiser. The application of electronic computers to factor analysis. *Educational and psychological measurement*, 20(1):141–151, 1960.

J. Lin, J. Dong, X. Dou, Y. Liu, P. Yang, and T. Ma. Psychological insights for incentive-based demand response incorporating battery energy storage systems: A two-loop stackelberg game approach. *Energy*, 239:122192, 2022.

Y. Long, D. Guan, K. Kanemoto, and A. Gasparatos. Negligible impacts of early covid-19 confinement on household carbon footprints in japan. *One Earth*, 4(4):553–564, 2021.

H. Lopes and M. West. Bayesian model assessment in factor analysis. *Statistica Sinica*, 14(1): 41–68, 2004.

M. C. Ruiz-Abellón, L. A. Fernández-Jiménez, A. Guillamón, A. Falces, A. García-Garre, and A. Gabaldón. Integration of demand response and short-term forecasting for the management of prosumers' demand and generation. *Energies*, 13(1):11, 2020.

F. Scharf and S. Nestler. Should Regularization Replace Simple Structure Rotation in Exploratory Factor Analysis? *Structural Equation Modeling*, 26(4):576–590, July 2019.

T. A. Schmitt and D. A. Sass. Rotation criteria and hypothesis testing for exploratory factor analysis: Implications for factor pattern loadings and interfactor correlations. *Educational and Psychological Measurement*, 71(1):95–113, Jan. 2011.

X. Shen, F. Tokoglu, X. Papademetris, and R. T. Constable. Groupwise whole-brain parcellation from resting-state fmri data for network node identification. *Neuroimage*, 82:403–415, 2013.

F. Wang, L. Liu, Y. Yu, G. Li, J. Li, M. Shafie-Khah, and J. a. P. Catalão. Impact analysis of customized feedback interventions on residential electricity load consumption behavior for demand response. *Energies*, 11(4):770, 2018.

A. Yates. *Multivariate Exploratory Data Analysis: A Perspective on Exploratory Factor Analysis.* State University of New York Press, USA, 1987. ISBN 0887065384.
